# Engineering MnPt Bimetallic Nanozymes for Cascade Enzymatic Therapy and Enhanced Radio‐Immunotherapy

**DOI:** 10.1002/advs.202519300

**Published:** 2026-03-12

**Authors:** Wenyi Zhang, Yangyang Guo, Guoping Xu, Luhaoxiang Liu, Lin Chen, Cai Zhang, Yiming Li, Jianmin Li, Yang Zhao

**Affiliations:** ^1^ Department of Radiology The Second Hospital of Tianjin Medical University Tianjin China; ^2^ Tianjin Institute of Urology The Second Hospital of Tianjin Medical University Tianjin China; ^3^ Department of Gastroenterology The Second Hospital of Tianjin Medical University Tianjin China

**Keywords:** cascade enzymatic activity, cGAS‐STING, immunogenic cell death, nanozyme, radioimmunotherapy

## Abstract

The immunosuppressive tumor microenvironment (TME) promotes resistance to radiation therapy, substantially limiting the efficacy of radioimmunotherapy. Here, we report a hyaluronic acid (HA)‐coated manganese–platinum (MnPt) bimetallic nanozyme (HD@MnO_2_) that integrates enzymatic cascade initiation with cGAS–STING pathway activation to facilitate radioimmunotherapy for breast cancer treatment. The HD@MnO_2_ features a cationic MnO_2_ core sequentially modified with HA and cisplatin, enabling CD44‐targeted delivery and improved biocompatibility. Leveraging the reversible valence cycling of Mn and Pt in the TME, HD@MnO_2_ offers multienzyme‐like activity, which collectively enhances H_2_O_2_ utilization, depletes glutathione, and promotes O_2_ generation. These cascade reactions combined with radiotherapy effectively trigger bursts of ROS production, disrupt redox homeostasis, and induce immunogenic cell death. Concurrently, released Mn^2^^+^ activates the cGAS–STING pathway, thereby boosting innate immunity. The combination of these effects promotes dendritic cell maturation and increases CD8^+^ T cell infiltration, thereby establishing a radiotherapy‐immune mutual amplification loop. Both in vitro and in vivo experiments demonstrated that HD@MnO_2_ markedly eliminates primary tumors and suppresses metastatic tumors by overcoming radioresistance and eliciting potent systemic antitumor immunity. Overall, this approach offers a promising strategy for addressing the conventional limitations of radioimmunotherapy.

## Graphical abstract

A CD44‐targeted MnPt bimetallic nanozyme (HD@MnO_2_) integrates multienzyme‐like activity with cGAS–STING activation to boost radio‐immunotherapy. Through cascade biocatalysis, it depletes glutathione, relieves hypoxia, and amplifies ROS, enhancing radiotherapy‐induced DNA damage and immunogenic cell death. The nanozyme promotes dendritic cell maturation and CD8^+^ T‐cell infiltration, effectively suppressing primary tumor growth and metastasis. This work offers a synergistic strategy to overcome radioresistance and elicit systemic antitumor immunity in breast cancer.

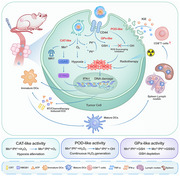

## 1 Introduction

Radiotherapy (RT) is a widely employed and effective cancer treatment that exerts cytotoxic effects through direct DNA damage and triggers immunogenic cell death (ICD) [1, 2, 3, 4]. For breast cancer, RT is accepted as a conventional therapeutic option [5, 6]. Postoperative RT is an essential noninvasive external therapy for eliminating microscopic tumor foci, which decreases the clinical mortality rate by reducing local recurrence [7]. However, the therapeutic outcomes of RT are often hindered by the immunosuppressive tumor microenvironment (TME) [8, 9]. Due to reduced reactive oxygen species (ROS) generation and DNA damage efficiency, hypoxic tumors exhibit enhanced radioresistance [10, 11, 12]. Additionally, the limited ICD‐induced antitumor immune response is insufficient for eliminating distant metastases, further restricting the clinical benefits of radioimmunotherapy [13, 14]. Therefore, strategies that can simultaneously modulate the TME, enhance ROS production, and activate antitumor immunity are crucial for improving RT efficacy.

To address these limitations, strategies targeting TME remodeling and immune activation have been explored [15, 16]. Hypoxia, elevated high hydrogen peroxide (H_2_O_2_) and glutathione (GSH) levels, and mild acidity are hallmark features of the TME [17, 18]. These qualities pose obstacles for conventional RT while also offering opportunities for TME triggered nanotechnology application. Nanozymes, a class of nanomaterials exhibiting enzyme‐like properties, have emerged as promising tools for tumor‐specific catalytic therapy [19, 20, 21, 22, 23]. Importantly, emerging evidence suggests that nanozymes can directly modulate antitumor immunity by triggering immunogenic cell death (ICD), promoting dendritic cell (DC) maturation, and activating the cGAS–STING pathway [24, 25, 26, 27, 28, 29]. Nanozymes with peroxidase (POD), catalase (CAT), oxidase, superoxide dismutase (SOD), and glutathione peroxidase (GPx)‐like activities can be broadly categorized based on their enzyme‐mimicking properties [24, 30]. Metal components often constitute the active sites of nanozymes, and since metals can accurately mimic the electronic redox processes that real enzymes catalyze, nanozymes benefit from multiplex, controlled, and sustained enzyme activities [31]. Therefore, well‐designed nanozymes combined with RT have the potential to alter TME and activate immunity for the effective and targeted killing of tumor cells [32, 33]. For instance, Mn‐based nanozymes reportedly alleviate hypoxia via CAT‐like activity and activate the cGAS‐STING pathway via Mn^2^^+^ release [34, 35, 36]; Pt‐based nanozymes exhibit CAT/POD‐like activity to generate ROS and sensitize cells to RT by enhancing radiation energy deposition [37, 38, 39]. However, monometallic nanozymes often suffer from single enzyme activity, insufficient TME regulation, and poor tumor targeting, limiting their therapeutic potential.

Bimetallic nanozymes, integrating the advantages of two metals, offer synergistic improvements in catalytic activity, TME responsiveness, and therapeutic efficacy. For instance, CuPt bimetallic nanozymes display significantly superior nanozyme catalytic activity [40]. FeMn bimetallic NPs have been engineered for enhanced POD‐like activity and potent radiosensitization [41]. The combination of Mn and Pt may potentially leverage Mn's redox cycling and STING‐activating properties with Pt's enzyme‐like and radiosensitizing capabilities. These features make MnPt bimetallic nanozymes ideal candidates for enhancing RT‐induced oxidative stress and reversing immunosuppression.

**SCHEME 1:**
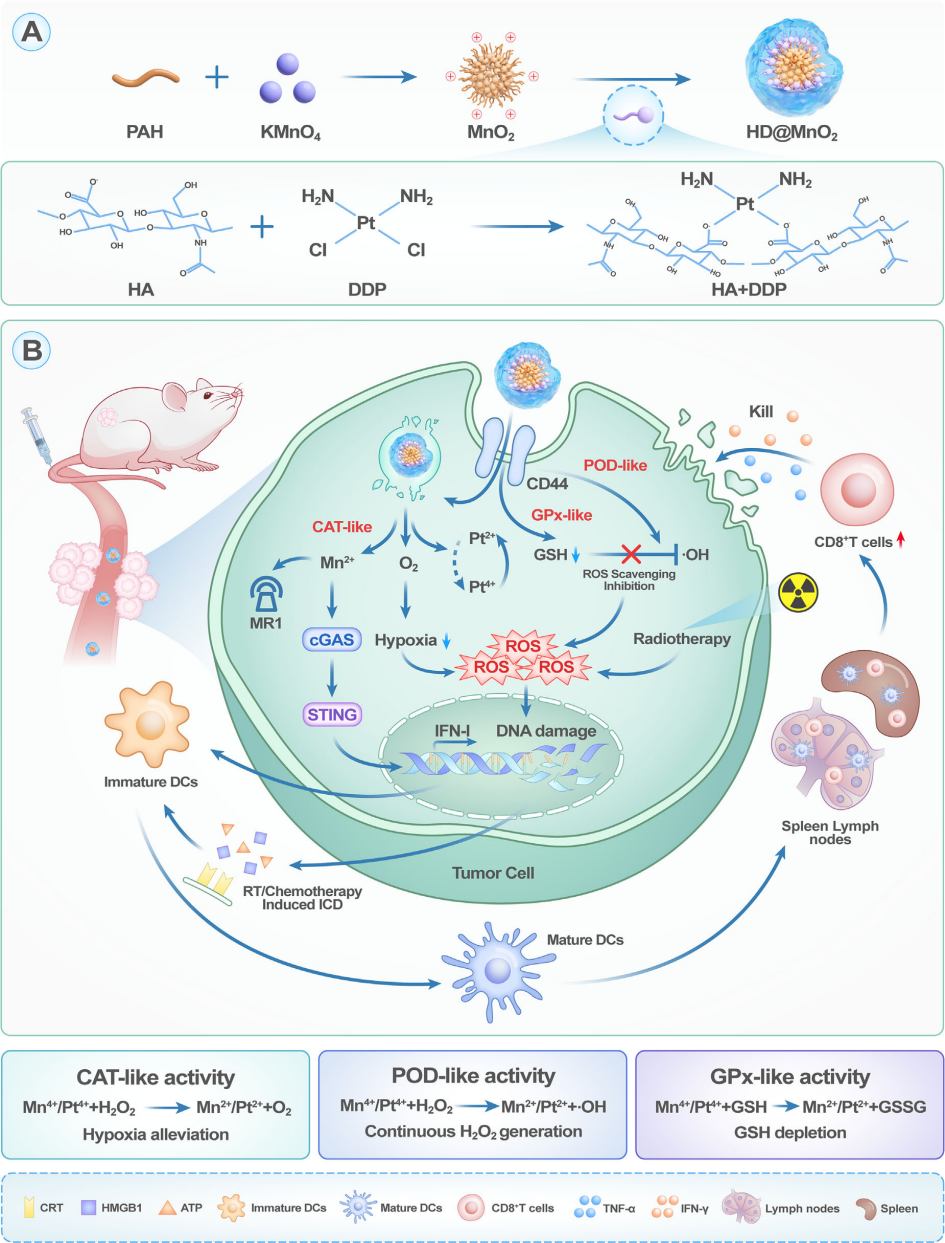
Schematic illustration of tumor‐targeted MnPt bimetallic nanozyme (HD@MnO_2_) for cascade enzymatic therapy and enhanced radioimmunotherapy in breast cancer.

In this study, we engineered a tumor‐targeted MnPt bimetallic nanozyme (HD@MnO_2_) through a facile and scalable self‐assembly process. Cationic MnO_2_ nanoparticles were first synthesized via the redox reaction between polyallylamine hydrochloride (PAH) and potassium permanganate, followed by surface functionalization with hyaluronic acid (HA) and cisplatin (DDP; Scheme 1A). HA enables CD44‐mediated tumor targeting and improves the biocompatibility of inorganic nanozymes, reducing clearance by the reticuloendothelial system (RES) and systemic toxicity [42]. The reversible valence cycling of Mn (Mn^2^^+^/Mn^4^^+^) and Pt (Pt^2^^+^/Pt^4^^+^) in the TME endows HD@MnO_2_ with multienzyme‐like activities, including POD‐like, CAT‐like, and GPx‐like activities, thereby enabling cascade enzyodynamic reactions to deplete GSH, alleviate hypoxia, and generate •OH. Rapid bursts of ROS and significant GSH depletion trigger apoptosis in tumor cells and enhance RT sensitivity (Scheme 1B). Moreover, HD@MnO_2_‐induced ICD promotes the maturation of dendritic cells (DCs) and the infiltration of CD8^+^ T cells. In parallel, the released Mn^2^^+^ ions activate the cGAS–STING signaling pathway and enhance the secretion of the inflammatory cytokine interleukin (IL)‐1β, further amplifying innate immune responses. Consequently, HD@MnO_2_ enhances the local efficacy of RT and elicits systemic antitumor immunity, effectively suppressing tumor metastasis. This work presents a rational design of a multifunctional nanozyme that integrates cascade biocatalysis, immune modulation, and radiotherapy, offering a promising strategy for overcoming the limitations of conventional radioimmunotherapy.

## 2 Results and Discussion

### 2.1 Synthesis and Characterization of HD@MnO_2_

Cationic manganese dioxide (MnO_2_) nanoparticles were synthesized via a redox reaction between KMnO_4_ and PAH under ambient conditions [43]. Subsequently, DDP and HA were sequentially added. Electrostatic interactions between the negatively charged HA and the positively charged MnO_2_ surface facilitated the self‐assembly of a core–shell nanostructure to form HD@MnO_2_ nanoparticles.

**FIGURE 1:**
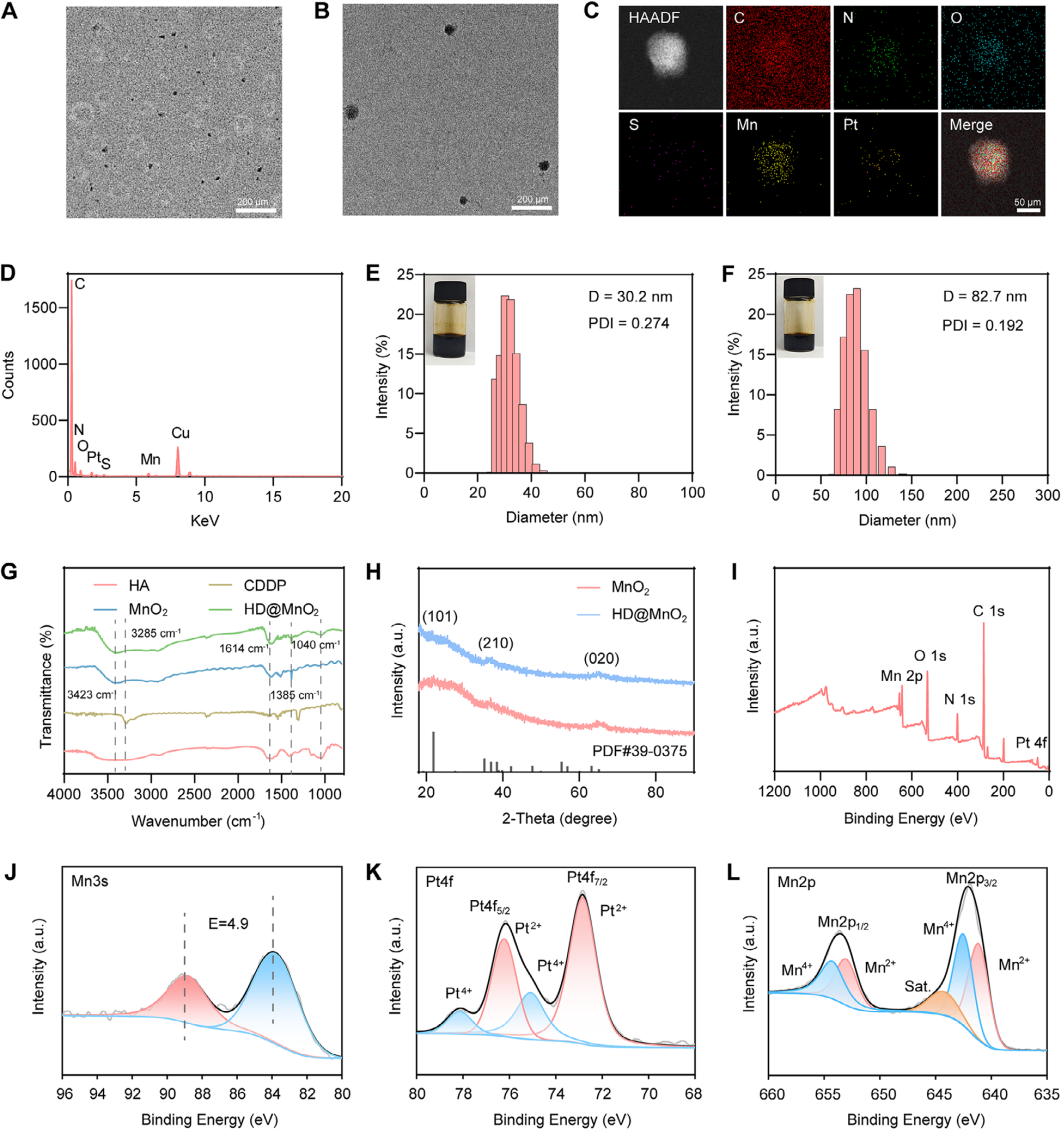
Preparation and characterization of the bimetallic nanozyme (HD@MnO_2_). A) Transmission electron microscopy (TEM) image of cationic manganese dioxide (MnO_2_)_,_ and B) HD@ MnO_2_ (scale bar: 200 µm). C) High‐angle annular dark‐field scanning transmission electron microscopy (HAADF‐STEM) and element mapping images of Mn, C, O, S, and Pt in the HD@MnO_2_. D) Energy‐dispersive X‐ray spectroscopy (EDX) of HD@MnO_2_. E) Hydrodynamic size distribution of MnO_2_ and F) HD@MnO_2_ assessed via dynamic light scattering (DLS) (PDI: polydispersity index). G) Fourier transform infrared spectroscopy (FT‐IR) of HD@MnO_2_. H) X‐ray diffraction (XRD) pattern of HD@MnO_2_ (JCPDS #39‐0375). I) X‐ray photoelectron spectroscopy (XPS) spectrum of HD@MnO_2_. J‐L) XPS spectrum of Mn 3s, Pt 4f, and Mn 2p of HD@MnO_2_.

To characterize the physical and electrochemical structure of the nanoparticled, transmission electron microscopy (TEM) was conducted, which revealed cationic MnO_2_ nanoparticles with a diameter of approximately 26.9 nm (Figure 1A). Measurements of electrical potential showed a strong positive surface charge (34.47 ± 1.50 mV), attributed to the PAH capping layer (Figure S1A). TEM revealed that HD@MnO_2_ exhibited a uniform spherical morphology with a diameter of approximately 55.0 nm after encapsulation with HA and DDP (Figure 1B). The surface charge reversed to −24.03 ± 1.90 mV, indicating successful electrostatic adsorption of the HA‐DDP layer onto the MnO_2_ surface (Figure S1A). The elemental distribution map revealed a uniform distribution of Pt and Mn, indicating successful integration of Pt into the manganese dioxide nanoparticles (Figure 1C). Energy‐dispersive X‐ray spectroscopy (EDX) was employed to analyze the elemental composition of HD@MnO_2_ (Figure 1D). The EDX spectrum confirmed characteristic peaks for C, N, O, Pt, S, Mn, and Cu. Dynamic light scattering (DLS) studies indicated an average hydrodynamic diameter of 30.2 nm and a PDI of 0.274 for MnO_2_ (Figure 1E), while for HD@MnO_2__,_ the diameter was 82.7 nm with a PDI of 0.192 (Figure 1F). This observation indicates that the diameter of MnO_2_ increased after coating with HA (Figure S1B), and the particle size became more uniform. We also found that the particle size of HD@MnO_2_ remained largely unchanged over a week during incubation in phosphate buffered saline (PBS), 10% fetal bovine serum (FBS) buffer and RPMI 1640 medium, demonstrating its excellent stability (Figure S2A,B).

To further characterize the chemical structure of HD@MnO_2_, Fourier transform infrared spectroscopy (FT‐IR) was conducted and it revealed characteristic peaks: at 3285 cm^−^^1^ for the O‐H peak of HA and at 1614 cm^−^^1^ for the C─O (COO^−^) peak (Figure 1G). X‐ray diffraction (XRD) was used to study the crystal structure of HD@MnO_2_. Diffraction peaks of HD@MnO_2_ and MnO_2_ were similar (Figure 1H), with the peaks indexed to the (101), (210), and (020) crystal planes of MnO_2_ (JPCDS #39‐0375). Next, X‐ray photoelectron spectroscopy (XPS) analysis was performed on freeze‐dried HD@MnO_2_. Five characteristic peaks were observed, corresponding to Mn 2p, O 1s, N 1s, C 1s, and Pt 4f, indicating the coexistence of HA, Pt, and MnO_2_ (Figure 1I). According to Mn 3s XPS spectra, the two Mn 3s peaks in the HD@MnO_2_ sample had a unique splitting energy (ΔE) of 4.9 eV, which is consistent with the distinctive oxidation state of Mn^4^^+^ (Figure 1J). Due to the coexistence of Pt^4^^+^ and Pt^2^^+^, the deconvolution of the HD@MnO_2_ XPS spectrum in the Pt 4f region revealed two distinctive peaks at 78.5 and 75.4 eV, corresponding to Pt^4^^+^ and Pt^2^^+^, respectively (Figure 1K). Due to the coexistence of Mn^4^^+^ and Mn^2^^+^, the deconvolution of the HD@MnO_2_ XPS spectra in the Mn 2p region revealed two distinctive peaks (Figure 1L). Subsequently, the loading rates of Mn and Pt during the synthesis of HD@MnO_2_ were determined, with an Mn loading rate of 72.80%±2.10% and a Pt loading rate of 63.62%±1.06% (Table S1). These findings strongly suggest that HD@MnO_2_ was successfully fabricated. To probe pH responsiveness, we tracked Mn^2+^ and DDP release at pH 7.4 and pH 5.6. Acidic conditions drove about 80–90% cumulative release within 3 days (Figure S3), confirming HD@MnO_2_’s acid‐triggered degradability, stability and biocompatibility for subsequent therapeutic investigations.

### 2.2 Multienzyme‐like Activity of HD@MnO_2_

**FIGURE 2:**
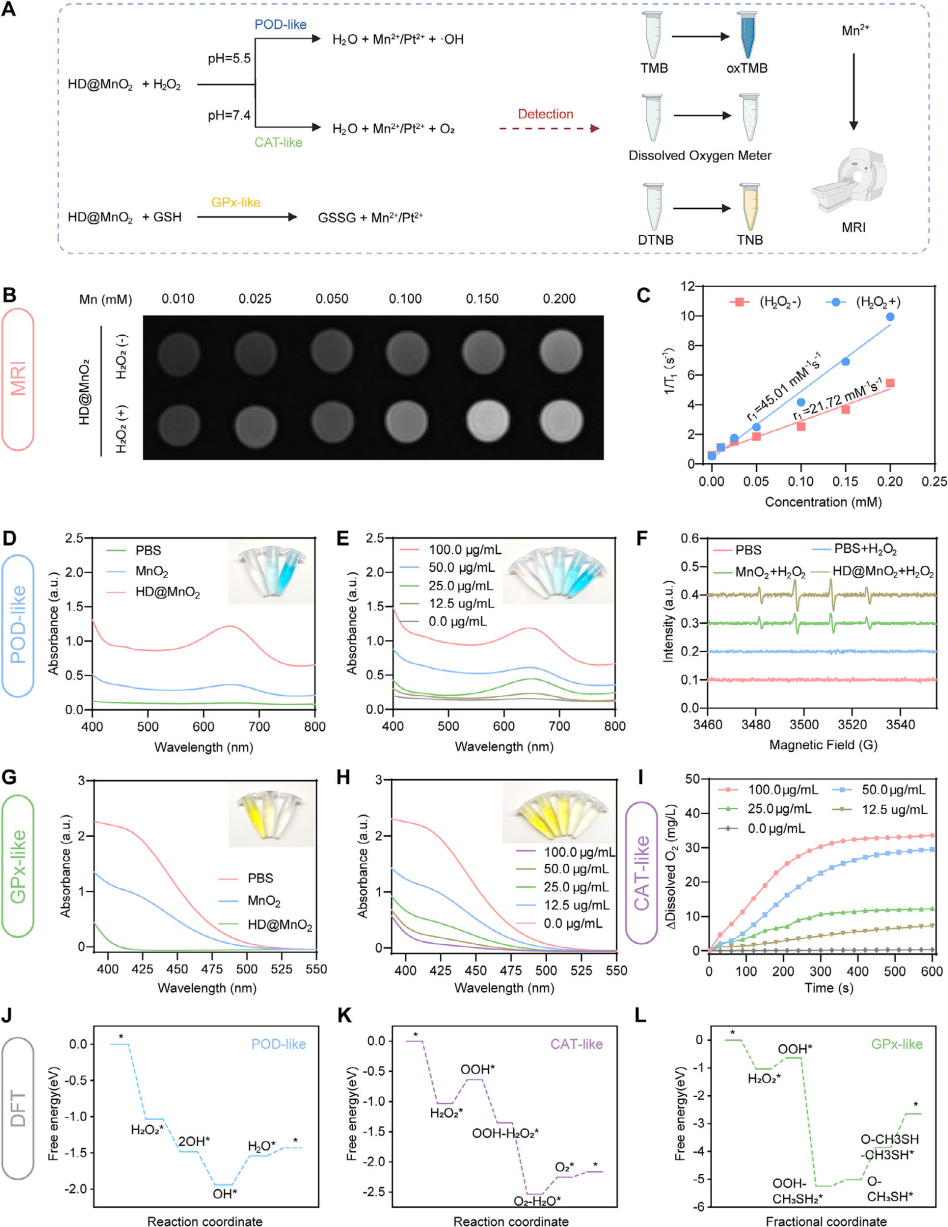
Multienzyme‐like activity of the bimetallic nanozyme (HD@MnO_2_). A) Schematic representation of the identification of multienzyme‐like activity of HD@MnO_2_. B) T_1_‐weighted magnetic resonance images of HD@MnO_2_. C) The longitudinal relaxation (r_1_) rates acquired from HD@MnO_2_. D‐E) Absorption spectra of the •OH generation process using tetramethylbenzidine (TMB) as a probe. F) Electron spin resonance (ESR) spectra of •OH generation in different groups by DMPO. G) The absorption spectra for detecting GSH depletion generated by various treatment conditions using 5,5‐dithiobis(2‐nitrobenzoic acid) (DTNB). H) HD@MnO_2_ concentration‐dependent glutathione (GSH) depletion. I) Dissolved oxygen curves of HD@MnO_2_ under different treatment conditions. J) Peroxidase (POD)‐like pathways of HD@MnO_2_ related to Gibbs free energy. K) Catalase (CAT)‐like pathways of HD@MnO_2_ related Gibbs free energy. L) Glutathion peroxidase (GPx)‐like pathways of HD@MnO_2_ related Gibbs free energy.

Considering that HD@MnO_2_ contains multivalent materials for nanocatalytic reactions, HD@MnO_2_ integrated enzymatic functionalities into a single platform, thus becoming a competitive multifunctional nanozyme. Importantly, the mutual reinforcement of these enzymatic processes led to the development of oxidative stress levels within the tumors (Figure 2). As an H_2_O_2_ enzyme‐mimicking catalyst, MnO_2_ has been reported to catalyze the decomposition of H_2_O_2_ into O_2_ or •OH (Figure 2A) [44]. First, we compared the H_2_O_2_ decomposition ability of HD@MnO_2_ in solutions. T_1_‐weighted magnetic renonance imaging (MRI) images showed that the T_1_ signal increased more significantly with increasing HD@MnO_2_ concentration in H_2_O_2_‐containing solutions compared to solutions without H_2_O_2_, indicating the release of more Mn^2+^ (Figure 2B,C). Furthermore, compared with the catalytic efficiency of MnO_2_ (Figure S4A,B), HD@MnO_2_ exhibited a higher catalytic efficiency, likely due to responsiveness of Mn and Pt to both H_2_O_2_ and GSH.

The enzyme‐mimetic activity of HD@MnO_2_ was examined in a buffer that simulates the tumor microenvironment (TME) conditions (pH 5.0; 10 mm GSH and 100 µm H_2_O_2_). To evaluate the POD‐like activity, we assessed •OH generation using tetramethylbenzidine (TMB) as a chromogenic probe. TMB reacts with •OH to form a blue oxidized product, which absorbs strongly at 652 nm [45]. Figure 2D illustrates that the unique absorption characteristics and blue color shift in the sample solution signify effective •OH production. Compared to MnO_2_, the HD@MnO_2_ group exhibited a higher absorption peak, which gradually increased with increasing HD@MnO_2_ concentration (Figure 2E). To further substantiate the catalytic effect of the POD‐like properties of HD@MnO_2_ nanoparticles, electron spin resonance (ESR) was employed to confirm the production of •OH [46]. Signals for •OH were observed in both the MnO_2_ and HD@MnO_2_ groups, with the latter showing a strong signal, indicating enhanced POD enzyme‐like activity (Figure 2F). Similarly, to investigate GPx‐like activity, we monitored GSH depletion using 5,5‐dithiobis(2‐nitrobenzoic acid) (DTNB). DTNB reacts with GSH to yield yellow TNB, which absorbs at 412 nm [47]. The results showed a significantly lower absorption peak and a faded color in the HD@MnO_2_ group than in the MnO_2_ group, indicating significant GSH reduction (Figure 2G). Furthermore, the absorption peak gradually decreased, and the solution color became lighter with increasing concentration of HD@MnO_2_ (Figure 2H), indicating its strong GPx‐like activity. To assess the CAT‐like activity of HD@MnO_2_, we investigated its capacity to breakdown H_2_O_2_ into oxygen. In H_2_O_2_ solution, the HD@MnO_2_ group showed a significant increase in dissolved oxygen over time compared to the MnO_2_ group (Figure S5). Similarly, increasing the HD@MnO_2_ concentration also led to a gradual increase in dissolved oxygen over the same time period (Figure 2I), demonstrating significant CAT‐like activity. In this study, we employed density functional theory (DFT) calculations to identify the dominant enzymatic activity among the multiple nanozyme functions (POD, CAT, and GPx) exhibited by HD@MnO_2_. The free‐energy difference (ΔG) of the reaction was used to evaluate the most probable reaction pathway and the rate‐determining step (RDS). DFT simulations further quantified the catalytic strengths of the three enzymes embedded in HD@MnO_2_. The RDS free energies for POD‐like, CAT‐like, and GPx‐like activities were determined to be 0.40, 0.40, and 1.20 eV, respectively (Figure 2J–L; Figure S6). Consequently, POD‐like and CAT‐like activities dominate in HD@MnO_2_, whereas GPx‐like activity is the least favorable. Benefiting from this synergistic multienzyme profile, HD@MnO_2_ represents a promising catalytic platform that can amplify ROS‐induced cell death and potentiate radioimmunotherapy. These findings indicate that HD@MnO_2_ possesses excellent POD/GPx/CAT‐like enzyme activities and is recognized as a multifunctional nanozyme.

### 2.3 Proliferation Inhibition and Radiosensitization In Vitro

**FIGURE 3:**
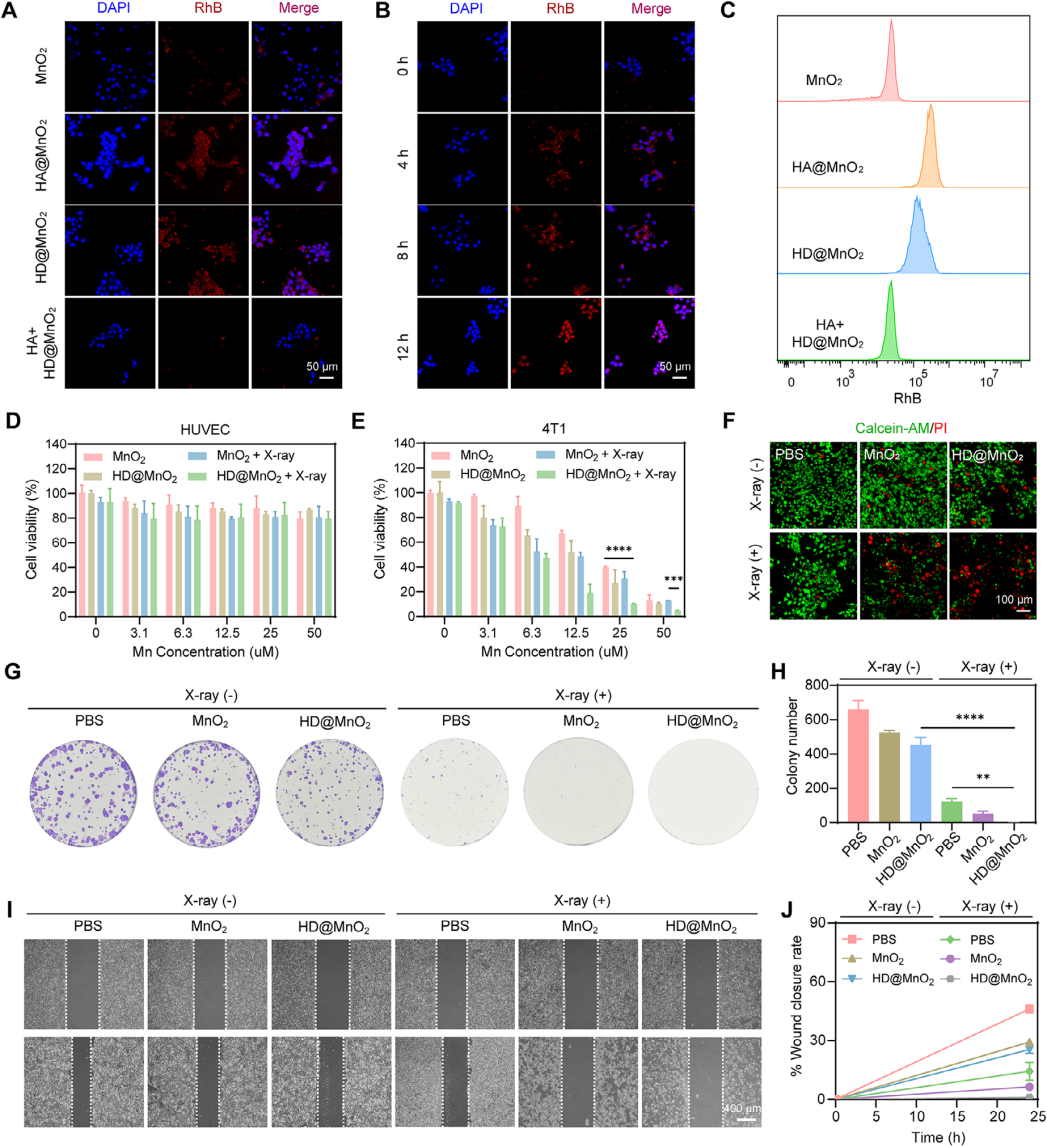
Cell internalization and in vitro therapeutic efficacy. A) Immunofluorescence (red) analysis of 4T1 cells treated with RhB‐HD@MnO_2_. B) Immunofluorescence (red) analysis of RhB‐HD@MnO_2_ uptake by 4T1 cells at different time points. C) Flow cytometry analysis of 4T1 cells treated with RhB‐HD@MnO_2_. Effects of HD@MnO_2_ on D) HUVECs and E) 4T1 cell viability in vitro, one‐way ANOVA with Tukey's post–hoc test, ^***^ *p* < 0.001, ^****^ *p* < 0.0001. F) Calcein‐AM/PI staining analysis of HD@MnO_2_‐induced apoptosis in 4T1 cells. G) Colony formation assay to determine the long‐term antitumor effect of HD@MnO_2_ and H) quantification of colony number, one‐way ANOVA with Tukey's post–hoc test, ^**^ *p* < 0.01, ^****^ *p* < 0.0001. I) Wound‐healing assay to determine the effect of HD@MnO_2_ on 4T1 cell migration and J) quantification of migration area.

Due to its significant multienzyme‐like properties, HD@MnO_2_ can be used to induce cancer cell death and enhance the efficacy of radiotherapy [48, 49, 50]. To theoretically validate the CD44‐targeting potential of HA, a key component of HD@MnO_2_, we first performed molecular docking simulations between HA and CD44 (Figure S7). The result revealed a binding affinity of ‐7.74 kcal/mol, a value that indicates a stable and specific intermolecular interaction, consistent with the well‐documented HA‐CD44 recognition motif. The targeting ability of HD@MnO_2_ was first verified by confocal laser scanning microscopy (CLSM) and flow cytometry analysis. First, MnO_2_, HA@MnO_2_ and HD@MnO_2_ were labeled with rhodamine B (RhB). As shown in Figure 3A,C, the HD@MnO_2_‐treated group exhibited a stronger red fluorescence signal than the MnO_2_ group. When 4T1 cells were pretreated with HA to block CD44 on their surface and then treated with HD@MnO_2_, the red fluorescence signal was significantly reduced, demonstrating that HD@MnO_2_ possesses robust CD44‐targeting ability. Additionally, the HD@MnO_2_‐treated group exhibited a fluorescence intensity slightly lower than that of the HA@MnO_2_ group (Figure S8A), but it was not statistically significant. These findings indicated that DDP grafting does not compromise the CD44‐targeting capability of HA and that efficient tumor‐specific recognition is preserved. Next, cellular uptake of HD@MnO_2_ was monitored at different time points and quantitatively analyzed. Overtime, the red fluorescence signal gradually increased, indicating that the cellular uptake efficiency of HD@MnO_2_ also increased (Figure 3B; Figure S8B).

Subsequently, the cytotoxicity of HD@MnO_2_ and MnO_2_ on HUVEC and 4T1 cells was tested. Neither HD@MnO_2_ nor MnO_2_ produced significant cytotoxicity in HUVEC at Mn concentrations ranging from 0 to 50 µm (Figure 3D). However, HD@MnO_2_ exhibited a stronger inhibitory effect on 4T1 cells than MnO_2_. Interestingly, a combined X‐ray and HD@MnO_2_ treatment reduced the viability of 4T1 cells to a minimum (Figure 3E). Furthermore, Calcein‐AM/PI staining revealed that HD@MnO_2_ induced cell death more strongly than MnO_2_, and combined X‐ray and HD@MnO_2_ treatment significantly increased the death rate of 4T1 cells further (Figure 3F).

To examine the long‐term inhibitory effect of HD@MnO_2_, a cell cloning assay was performed. Compared with MnO_2_, HD@MnO_2_ treatment reduced the colony‐forming ability of 4T1 cells (Figure 3G,H). Combined treatment with X‐ray and HD@MnO_2_ almost completely inhibited colony formation.

The poor prognosis of malignant tumors arises not only from rapid cell proliferation but also from strong metastatic potential [51, 52]. Therefore, we further investigated the effect of this nanozyme on the migration of 4T1 cells. A cell scratch experiment was employed to examine cell migration, where cells preincubated with different formulations were assessed post‐scratching to determine the inhibitory effect of HD@MnO_2_ on cell migration. HD@MnO_2_ showed a more pronounced inhibitory effect on scratch healing rates compared to MnO_2_, and the combination application of X‐ray and HD@MnO_2_ considerably impeded the migration of 4T1 cells (Figure 3I,J). These results demonstrated that this nanozyme possesses both excellent tumor‐suppressive and radiosensitizing properties.

### 2.4 HD@MnO_2_ Enhances ROS Generation, DNA Damage and ICD to Augment Radiosensitization

**FIGURE 4:**
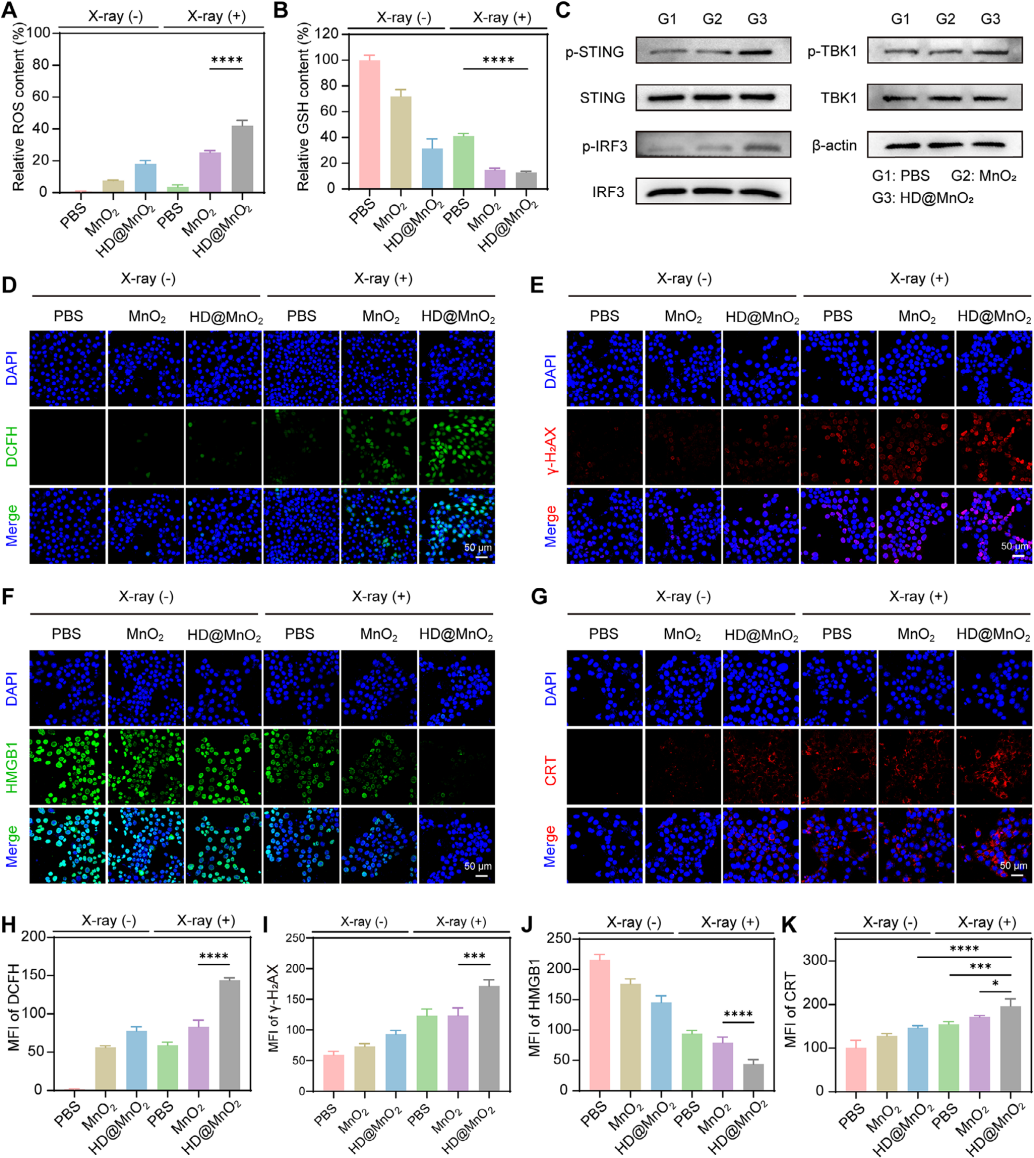
HD@MnO_2_ in vitro enhanced Radiosensitization. A) Quantification of ROS levels in 4T1 cells with various treatments using flow cytometry, ANOVA with Tukey's post–hoc test, *p*‐values: ^**^ *p* < 0.01, ^***^ *p* < 0.001, ^****^ *p* < 0.0001. B) Relative GSH levels in 4T1 cells with various treatments, ANOVA with Tukey's post–hoc test, *p*‐values: ^**^ *p* < 0.01, ^***^ *p* < 0.001, ^****^ *p* < 0.0001. C) Western blot analysis of the expression of cGAS‐STING signaling molecules in 4T1 cells after different treatments. D) Intracellular ROS levels in 4T1 cells were detected using a DCFH‐DA probe (green). E) γ‐H_2_AX, F) HMGB1, and G) CRT levels in 4T1 cells were assessed using immunofluorescence. Quantitative analysis of mean fluorescence intensity (MFI) of H) ROS, I) γ‐H2AX, J) HMGB1, and K) CRT was performed and analyzed using one‐way ANOVA with Tukey's post–hoc test, ^*^ *p* < 0.05, ^***^ *p* < 0.001, ^****^ *p* < 0.0001.

Recent investigations have shown that ROS can trigger tumor cell death and augment radiosensitization through the induction of DNA damage. To further explore the anti‐tumor and radiosensitization mechanisms of HD@MnO_2_, we first verified ROS generation using the 2′,7′‐dichlorodihydrofluorescein diacetate (DCFH‐DA) probe, which emits green fluorescence in the presence of ROS. Flow‐cytometric and immunofluorescence analyses revealed that HD@MnO_2_ elicited markedly stronger green fluorescence than MnO_2_ alone (Figure 4A,D,H), likely due to increased cellular uptake of HD@MnO_2_ and its more robust mutienzyme catalytic system. Furthermore, the fluorescence intensity of the combined X‐ray and HD@MnO_2_ group was significantly higher than that of the other groups, indicating a significant increase in ROS generation.

Subsequently, the GSH level of the treated 4T1 cells was measured, as shown in Figure 4B. The HD@MnO_2_ + X‐ray group decreased the cellular GSH level. We further investigated whether these stress responses could activate innate immune signaling within tumor cells. Given that the cGAS–STING axis serves as a critical DNA‐sensing pathway linking genotoxic stress to immune activation, we first evaluated its status in 4T1 cells. Western blot analysis revealed that the HD@MnO_2_ treatment markedly up‐regulated the phosphorylation levels of STING, TBK1, and IRF3 (Figure 4C), thus demonstrating robust activation of the STING signaling cascade at the tumor‐cell level. Since radiotherapy can directly cause DNA damage, γ‐H_2_AX staining was performed in all treatment groups. The MnO_2_ group showed only weak red fluorescence, while the HD@MnO_2_ group showed a slight increase in the red fluorescence signal (Figure 4E,I). The red fluorescence intensity of the combined (X‐ray and HD@MnO_2_) group was significantly enhanced, indicating that the degree of DNA damage caused to 4T1 cells was the most significant under this treatment.

The efficacy of radiotherapy is often limited by inefficient ICD [53, 54]. This study examined whether HD@MnO_2_ could enhance the in vitro induction of ICD by measuring HMGB1 secretion, CRT exposure and release of ATP in each treatment group. The nuclear HMGB1 green fluorescence signal in the MnO_2_ group was slightly lower than that in the PBS group, while the HD@MnO_2_ group exhibited even lower green fluorescence than the MnO_2_ group. Furthermore, the fluorescence intensity in the combined X‐ray and HD@MnO_2_ group was significantly lower than that in the other groups (Figure 4F). This phenomenon was further corroborated by quantitative mean fluorescence intensity (MFI) measurements (Figure 4J). Moreover, CLSM images (Figure 4G) and MFI data (Figure 4K) revealed that the HD@MnO_2_ group exhibited higher CRT exposure on the cell membrane than the MnO_2_ group. Notably, the combined X‐ray and HD@MnO_2_ group exhibited significantly higher CRT fluorescence levels than the other groups. Similarly, HD@MnO_2_ increased ATP release more than MnO_2_, and when X‐ray and HD@MnO_2_ were combined, ATP release reached its maximum (Figure S9). These results indicate that HD@MnO_2_ triggers ROS accumulation through nanocatalytic reactions, leading to increased DNA damage and enhanced ICD. This, in turn, promotes the activation of antitumor immune responses.

### 2.5 HD@MnO_2_ Enhances Radiotherapy through Dendritic Cell (DC) Maturation and T Cell Activation

**FIGURE 5:**
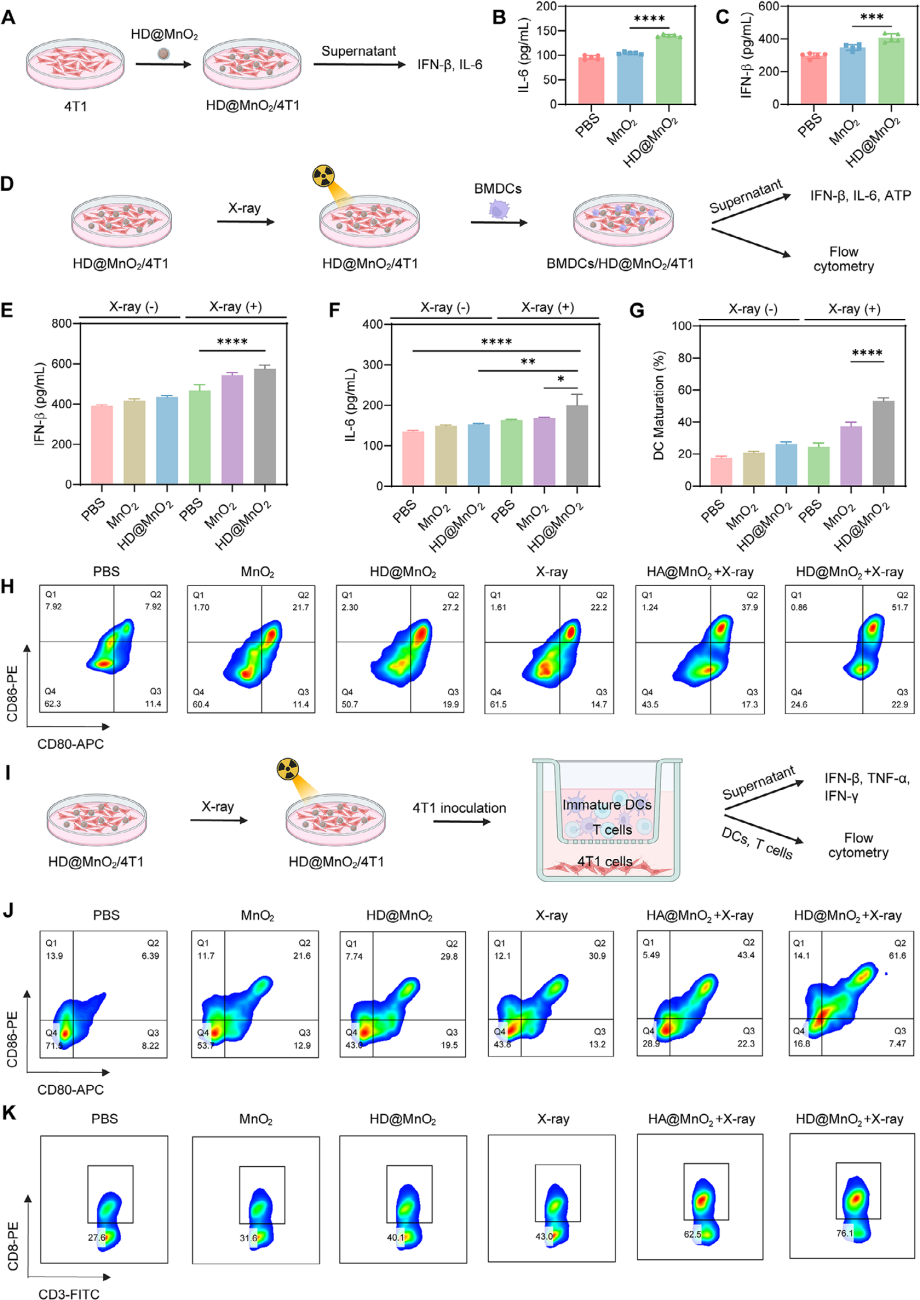
HD@MnO_2_ induces anti‐tumor immunity in vitro. A) Cytokine analysis process in the supernatant after HD@MnO_2_ treatment, including B) IL‐6 and C) IFN‐β, ANOVA with Tukey's post–hoc test, ^***^ *p* < 0.001, ^****^ *p* < 0.0001. D) Cytokine analysis process in the supernatant after X‐ray combined with HD@MnO_2_ co‐culture, including E) IFN‐β and F) IL‐6, and G) Quantitative analysis of DC maturation, ANOVA with Tukey's post–hoc test, ^*^ *p* < 0.05, ^**^ *p* < 0.01, ^****^ *p* < 0.0001. H) Flow cytometry analysis of DC maturation. I) The Transwell system was utilized to explore the maturation of DCs and their interaction with T cells induced by different treatments for 4T1 cells. The upper layer is DCs and T cells, and the lower layer is 4T1 cells. J) Flow cytometry analysis of BMDCs maturation induced by the culture medium supernatant of 4T1 cells. K) CD8^+^ T cells were induced by the culture medium supernatant of 4T1 cells pretreated from different treatments.

Dendritic cell (DC) maturation is crucial for the initiation and maintenance of both innate and adaptive immunity [55, 56]. Mature DCs secrete proinflammatory cytokines, including interferon‐β (IFN‐β), tumor necrosis factor‐α (TNF‐α), and interleukin‐6 (IL‐6). These cytokines stimulate T cell proliferation and differentiation, thereby activating antitumor immune responses [57, 58]. To evaluate the immunostimulatory effect of HD@MnO_2_, we first assessed its influence on cytokine secretion by 4T1 cells (Figure 5A). While MnO_2_ induced only a slight increase in IFN‐β and IL‐6, HD@MnO_2_ induced more robust secretion of all of these cytokines (Figure 5B,C).

Next, we established a co‐culture system and observed DC maturation in vitro following X‐ray treatment (Figure 5D). We evaluated the ability of HD@MnO_2_ to promote DC maturation. After co‐culturing of 4T1 cell with BALB/c mouse bone marrow‐derived DCs, cytokine levels in the co‐culture suspensions were assessed (Figure 5E,F). The combination treatment of X‐ray and HD@MnO_2_ elicited the highest levels of IFN‐β and IL‐6 secretion. Flow cytometry demonstrated that MnO_2_ alone elevated the percentage of mature DCs from 18.7% to 21.7%, indicating a weak immune activation effect. However, HD@MnO_2_ increased the proportion of mature DCs to 27.2%. Combined X‐ray and HD@MnO_2_ significantly enhanced DC maturation, reaching 51.7%, which represents a 2.76‐fold increase relative to the control group (Figure 5G,H).

To determine whether matured DCs could relay immunogenic signals to T cells and achieve tumoricidal synergy, we built a Transwell‐based in vitro co‐culture system (Figure 5I). 4T1 cells subjected to different treatments were confined to the lower chamber, while bone‐marrow‐derived DCs and syngeneic splenic T cells were co‐cultured in the upper chamber. As shown in Figure S10, the HD@MnO_2_ + X‐ray group yielded a marked increase of IFN‐β, IFN‐γ and TNF‐α in the supernatant as detected with an ELISA, indicating that immunogenic signalling was translated into genuine T‐cell effector output. Concomitantly, flow cytometry showed that expression of CD80 and CD86 rose from 6.39 % in untreated 4T1 cells to 61.61 % under the HD@MnO_2_ + X‐ray treatment (Figure 5J; Figure S11), and the CD8^+^ T cells in the upper chamber expanded proportionally (Figure 5K; Figure S11). These data suggested that HD@MnO_2_‐mediated nanocatalysis not only induces robust ICD and effectively promotes DC maturation, but also potentially sensitizes radiotherapy and triggers potent antitumor immune responses.

Notably, HD@MnO_2_ functions as a broad‐spectrum radiosensitizer. Its radio‐sensitizing efficacy stems from intrinsic cascade nanozyme activity that amplifies radiotherapy‐induced ROS production, depletes intracellular GSH, and alleviates tumor hypoxia. Additionally, the released Mn^2^^+^ from HD@MnO_2_ activates the cGAS–STING pathway to potentiate innate immunity, further reinforcing the synergy with radiotherapy without relying on specific irradiation wavelengths. This unique mechanism enables HD@MnO_2_ to exert radio‐sensitizing effects across different tumor types and radiotherapy settings, highlighting its translational potential in clinical applications.

### 2.6 Antitumor Effect In Vivo

**FIGURE 6:**
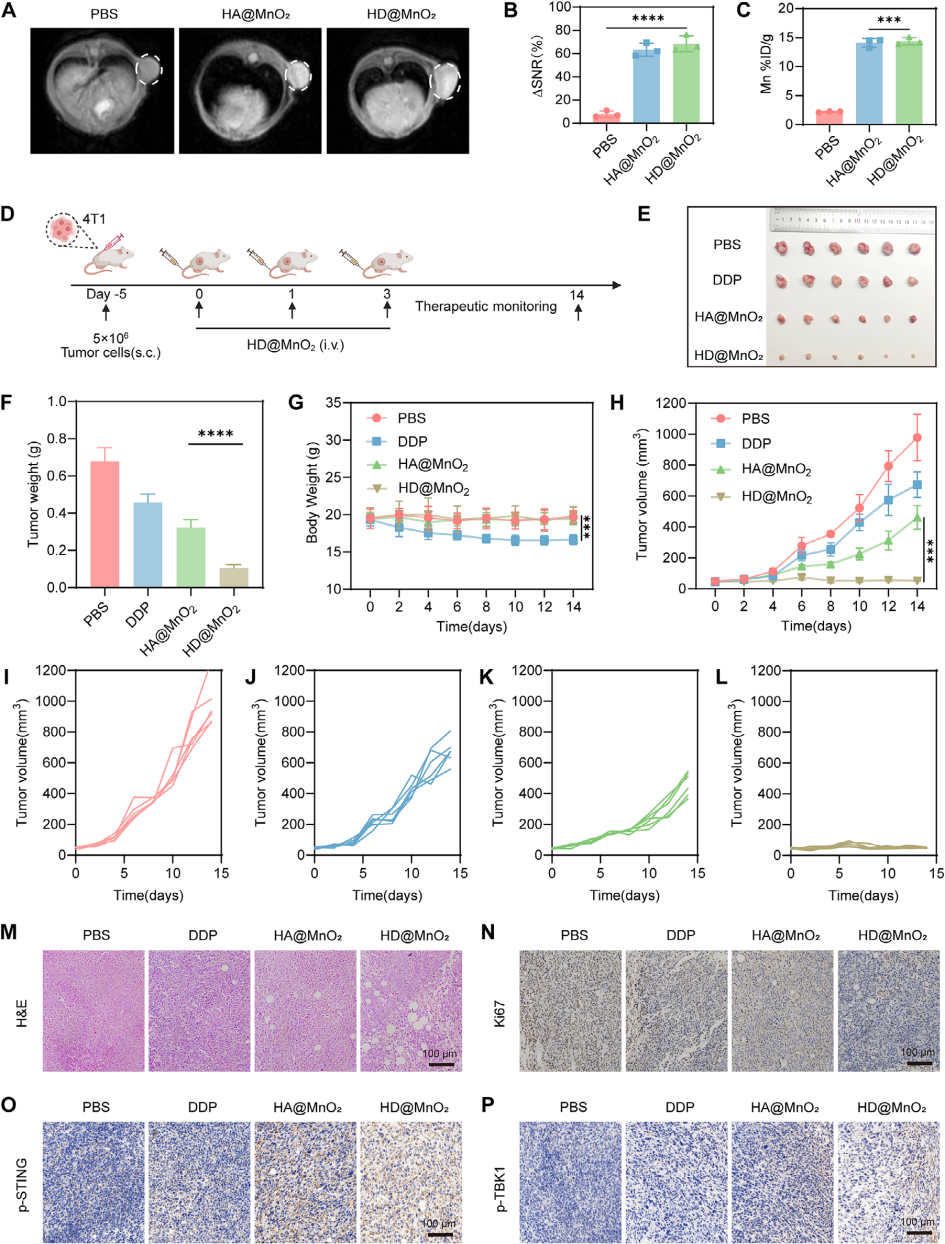
In vivo imaging and therapeutic effects of HD@MnO_2_. A) T_1_‐weighted MR images captured before and after the injection of MnO_2_ and HD@MnO_2_. B) The corresponding ΔSNR at axial of MnO_2_ and HD@MnO_2_, ANOVA with Tukey's post–hoc test, ^****^ *p* < 0.0001. C) Quantification of Mn content in tumor tissue, ANOVA with Tukey's post–hoc test, ^***^ *p* < 0.001. D) Protocol of subcutaneous tumor model establishment and treatment process. E) Visual documentation of tumor‐bearing mice post‐intravenous administration of several formulations. F) Tumor weight. G) Trends in body weight alterations for each experimental group, ANOVA with Tukey's post–hoc test, *p*‐values: ^**^ *p* < 0.01, ^***^ *p* < 0.001, ^****^ *p* < 0.0001 (n = 6). H) Tumor progression curves for each mouse cohort, ANOVA with Tukey's post–hoc test, *p*‐values: ^**^ *p* < 0.01, ^***^ *p* < 0.001, ^****^ *p* < 0.0001 (n = 6), I‐L) Detailed tumor growth trajectories for individual treatment regimens (n = 6), I) PBS group, J) DDP group, K) HA@MnO_2_ group, L) HD@MnO_2_ group. M) H&E staining, N) Ki67, O) p‐STING and P) p‐TBK1 immunohistochemistry of tumor tissue.

Based on the strong therapeutic efficacy and immunostimulatory effects of HD@MnO_2_ observed in vitro, we next investigated its antitumor potential in vivo. First, we evaluated the imaging performance of HD@MnO_2_ in tumors in a 4T1 subcutaneous tumor‐bearing mouse model. Since direct injection of MnO_2_ is highly toxic [59], MnO_2_ was encapsulated with HA to create HA@MnO_2_. HD@MnO_2_ and HA@MnO_2_ demonstrated comparable T_1_‐weighted signal enhancement in tumor tissue (Figure 6A), as confirmed by analysis of the change in MRI signal‐to‐noise ratio (ΔSNR) (Figure 6B). Quantitative analysis of Mn content in tumor tissue revealed no significant difference between the HD@MnO_2_ and HA@MnO_2_ groups, demonstrating successful tumor accumulation of the nanozyme (Figure 6C). In addition, we tested the Mn content in various organs at different time points. The results showed that Mn primarily accumulated in the liver and kidneys, with its content reaching a peak at 4 h post‐injection, which was largely cleared after 48 h (Figure S12). We then tested the therapeutic efficacy of HA@MnO_2_ in vivo. When tumors grew to approximately 100 mm^3^, mice were randomly divided into four groups: PBS, DDP, HA@MnO_2_, and HD@MnO_2_. The corresponding drugs were injected on days 0, 1, and 3. Tumor growth was subsequently monitored (Figure 6D). The results showed that the HD@MnO_2_ group had smaller tumors than the other groups, likely due to the catalytic activity of the dual nanozymes in HD@MnO_2_ (Figure 6E). On day 14 of treatment, mice were euthanized, and tumors were excised and weighed (Figure 6F). The results were consistent with the tumor growth curves (Figure 6H–L). Before euthanization, mice were weighed on alternate days (Figure 6G). The DDP group demonstrated a notable reduction in body weight, ascribed to the deleterious effects of chemotherapy. No notable disparities in body weight were detected among the remaining groups. Quantitative analysis of alanine transferase (ALT), aspartate transferase (AST), creatinine (CREA), and urease (UREA) showed that HD@MnO_2_ treatment did not significantly affect liver and kidney function (Figure S13), indicating that HD@MnO_2_ is well tolerated. The hematoxylin and eosin (H&E) staining of essential organs, including the heart, liver, spleen, lungs, kidneys, and brain, across all treatment groups revealed no significant pathological changes (Figure S14). Additionally, cell proliferation was evaluated using hematoxylin and eosin (H&E) staining and Ki67 staining, thereby corroborating the therapeutic efficacy of HD@MnO_2_ (Figure 6M,N). Given its potent tumor‐suppressive capacity, we further asked whether HD@MnO_2_ elicits innate immune signaling in vivo. Immunostaining of tumor sections revealed pronounced up‐regulation of p‐STING and p‐TBK1 (Figure 6O,P), thereby confirming activation of the cGAS‐STING pathway within the TME.

### 2.7 Radiosensitization and Immune Stimulation In Vivo

**FIGURE 7:**
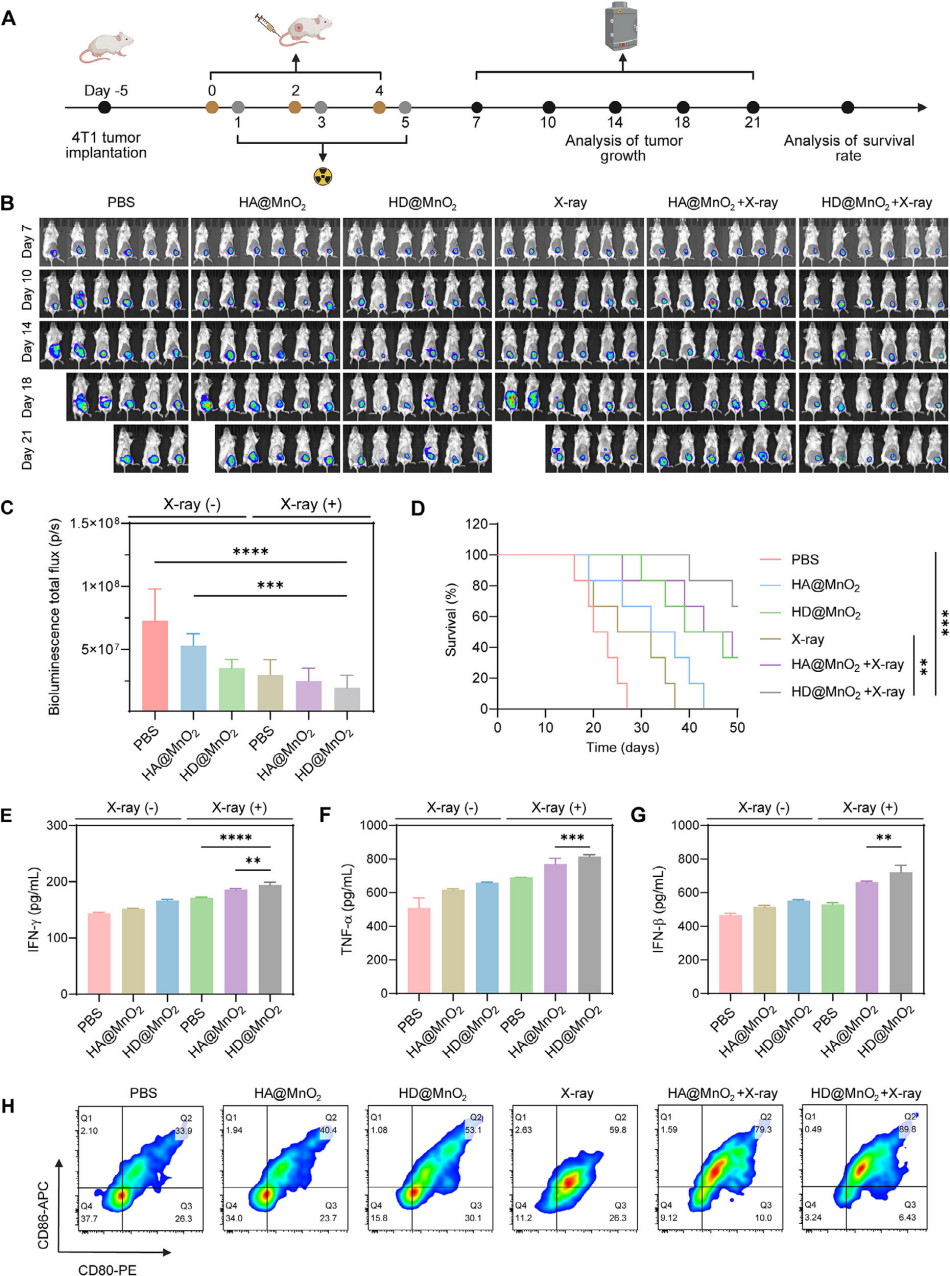
HD@MnO_2_ exerts radiosensitization effects in vivo by activating anti‐tumor immunity. A) Scheme of 4T1 orthotopic tumor establishment and treatment schedule. B) In vivo BLIs of 4T1 tumor‐bearing mice and C) quantification analysis of 4T1 tumor bioluminescence (BLI) signals (n = 6). D) Survival curves of 4T1 tumor‐bearing mice, ANOVA with Tukey's post–hoc test, p‐values: ^**^ *p* < 0.01, ^***^ *p* < 0.001, ^****^ *p* < 0.0001 (n = 6). Quantification of E) IFN‐γ, F) TNF‐α, and G) IFN‐β in tumor tissue, ANOVA with Tukey's post–hoc test, *p*‐values: ^**^ *p* < 0.01, ^***^ *p* < 0.001, ^****^ *p* < 0.0001 (n = 3). H) Representative flow cytometry plots depicting CD80^+^/CD86^+^ DC maturation in 4T1 tumors.

Based on the radiosensitization and immunostimulatory effects of HD@MnO_2_ in vitro, this study further evaluated its corresponding effects in vivo. Orthotopic 4T1‐luc tumor‐bearing mice were subsequently established to replicate the normal development of human breast cancer [60]. In the combination treatment group, 4T1‐luc tumor‐bearing mice received intravenous injections of HD@MnO_2_ on days 0, 2, and 4 and were exposed to X‐ray irradiation on days 1, 3, and 5 (Figure 7A). We administered a low dose (4 Gy) of irradiation 6 h postintravenous injection to minimize adverse effects on normal tissues. In vivo bioluminescence (BLI) revealed that the HD@MnO_2_ group exhibited a lower fluorescence flux than the HA@MnO_2_ group, irrespective of X‐ray irradiation. Consistent with the in vitro results, the combined application of X‐ray and HD@MnO_2_ exhibited the greatest tumor inhibition effect (Figure 7B,C). Moreover, survival analysis demonstrated that mice receiving combined X‐ray and HD@MnO_2_ treatment had significantly prolonged survival relative to the other groups (Figure 7D).

To further evaluate the immunostimulatory effects of this combined therapy in vivo, we systematically investigated the immune responses following different treatment regimens. The results showed that, under X‐ray irradiation, HD@MnO_2_ treatment elicited higher secretion of the immune mediators IFN‐γ (Figure 7E), TNF‐α (Figure 7F), and IFN‐β (Figure 7G) than the MnO_2_ treatment. Flow cytometric analysis of cells labeled for CD80 and CD86 revealed that the combined X‐ray and HD@MnO_2_ group increased the proportion of mature dendritic cells from 33.9% to 89.8%, markedly surpassing the proportions observed in the other groups (Figure 7H; Figure S16A). CD8^+^ T cells are the key effector cells in anti‐tumor immunity. Therefore, we further analyzed the CD8^+^ T cell content in each group. The results revealed that MnO_2_ treatment only increased the CD8^+^ T cell ratio from 20.2% to 22.7%, while HDM increased the ratio to 27.7%. Notably, combined X‐ray and HD@MnO_2_ treatment significantly increased this ratio further to 36.0% (Figure S15 and S16B). Together, these results indicate that HD@MnO_2_ markedly enhanced radiosensitization and activated antitumor immune responses in vivo.

### 2.8 HD@MnO_2_‐Based Radio‐Immunotherapy Restrains 4T1‐luc Lung Metastasis

**FIGURE 8:**
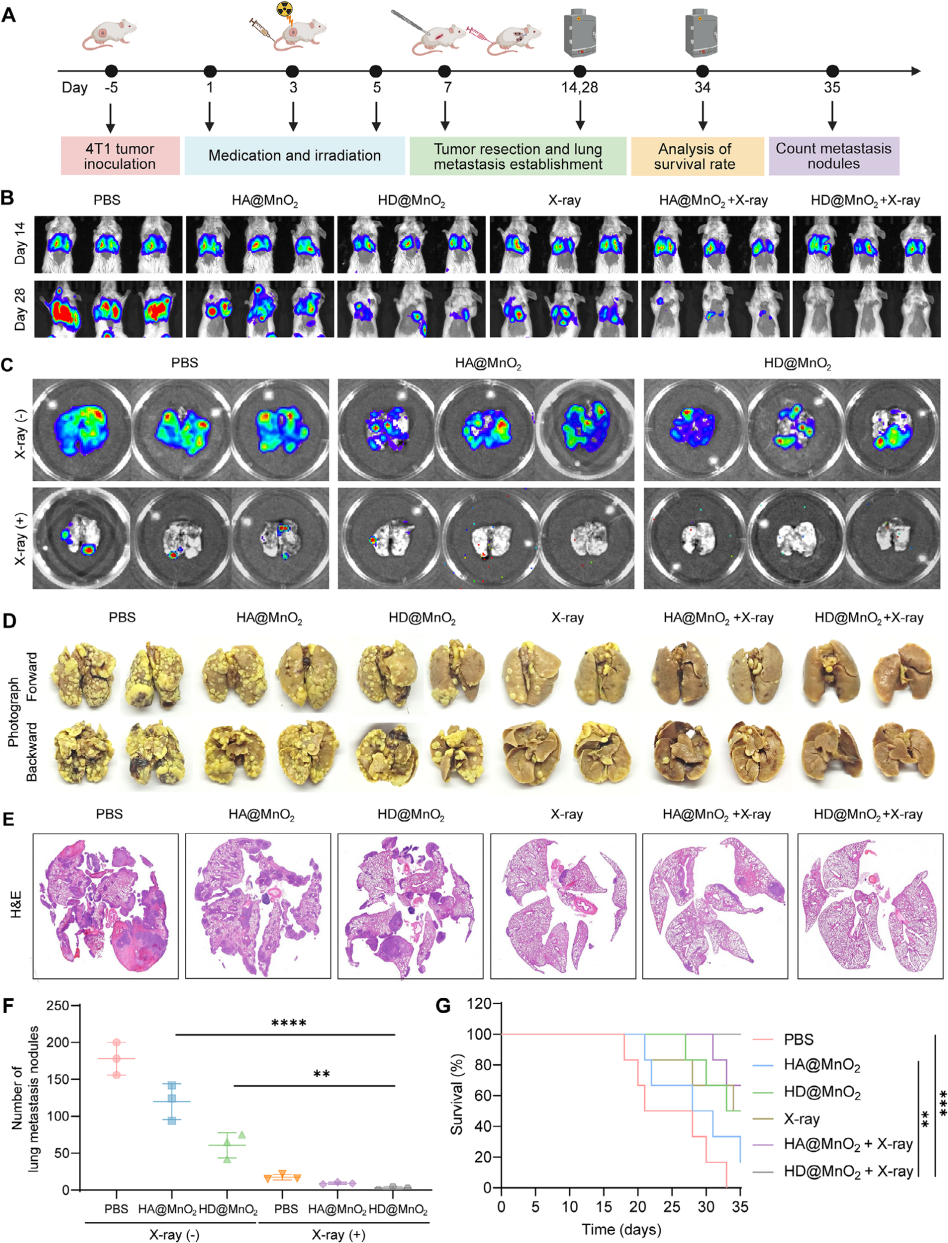
HD@MnO_2_ effectively inhibits 4T1‐luc lung metastasis. A) Schematic illustration of lung metastasis tumor‐bearing mouse model establishment and treatment schedule. B) Representative bioluminescence images of metastatic lesions in 4T1‐luc tumor‐bearing mice after different treatments (n = 3). C) Representative bioluminescence images of lung metastatic lesions ex vivo (n = 3). D) Representative images of the Bouin's Fixative‐stained whole lungs. E) Representative H&E‐stained lung tissue sections. F) Quantitative analysis of metastasis nodules with different treatments, ANOVA with Tukey's post–hoc test, *p*‐values: ^**^ *p* < 0.01, ^***^ *p* < 0.001, ^****^ *p* < 0.0001. G) Kaplan–Meier survival curves, ANOVA with Tukey's post–hoc test, *p*‐values: ^**^ *p* < 0.01, ^***^ *p* < 0.001, ^****^ *p* < 0.0001 (n = 6).

To determine whether HD@MnO_2_‐elicited systemic immunity curbs postsurgical tumor metastasis, we resected primary 4T1‐luc tumors five days after engraftment and immediately injected 4T1‐luc tumor cells via the tail vein to establish a lung‐only metastasis model Figure 8A). Mice then received three i.v. doses of HD@MnO_2_ followed by local X‐ray post‐injection. By day 28, bioluminescence of pulmonary lesions in the HD@MnO_2_ + X‐ray group was lower than that of the PBS or X‐ray alone groups (Figure 8B). Ex vivo imaging of excised lungs corroborated these data, showing discrete luciferase foci in controls but only background signal in the combination group (Figure 8C). Bouin's staining revealed 178 ± 22 surface nodules in PBS‐treated mice, which reduced to 3 ± 2 after HD@MnO_2_ + X‐ray treatment (Figure 8D,F). Histology on day 35 showed large proliferative lesions in controls, whereas HD@MnO_2_ + X‐ray lungs retained >95 % normal alveolar architecture (Figure 8E). Kaplan–Meier analysis revealed prolonged survival with the combination treatment (Figure 8G), without weight loss or fibrosis. Thus, HD@MnO_2_‐mediated radio‐immunotherapy converts the lung microenvironment from metastasis‐permissive to metastasis‐resistant.

## 3 Conclusion

In summary, we developed a tumor‐targeted MnPt bimetallic nanozyme that integrates cascade biocatalysis, immune modulation, and radiotherapy into a single nanoplatform for synergistic radio‐immunotherapy. By leveraging the cascade enzymatic activity (POD, CAT, GPx) of Mn and Pt, HD@MnO_2_ efficiently modulates the TME through GSH depletion, hypoxia relief, and ROS generation, thereby enhancing radiotherapy‐induced DNA damage and overcoming radioresistance. Importantly, the nanozyme‐induced immunogenic cell death promoted dendritic cell maturation and CD8^+^ T cell infiltration, while the released Mn^2^^+^ ions activated the cGAS–STING pathway, amplifying innate immune responses. This dual mechanism not only enhances local tumor control but also elicits systemic antitumor immunity, effectively suppressing metastasis and extending survival. Despite these promising results, several translational considerations should be noted. While the in vivo biosafety profile appeared favorable under tested conditions, potential long‐term or dose‐dependent toxicity related to Mn accumulation in clearance organs warrants further investigation. Additionally, the reliance on CD44 targeting may face challenges in tumors with heterogeneous receptor expression. Future studies could explore combination strategies, such as coupling HD@MnO_2_ with immune checkpoint inhibitors (e.g., anti‐PD‐1/PD‐L1), to further enhance adaptive immunity and overcome therapeutic resistance. Moreover, scaling up the synthesis with batch‐to‐batch consistency and ensuring long‐term stability remain important steps toward clinical translation. Collectively, this work presents a rational design of a multifunctional nanozyme that addresses key limitations of conventional radioimmunotherapy, offering a promising and adaptable strategy for breast cancer treatment with the potential to induce durable immune memory.

## 4 Experimental Section

### 4.1 Ethics Statement

The animal study was performed in line with the National Institutes of Health Guide for the Care and Use of Laboratory Animals and approved by the Animal Ethics Committee of Tianjin University of Traditional Chinese Medicine (approval number: TCM‐LAEC2023104).

### 4.2 Statistical Analysis

All the experimental data were repeated three or more times, and each experiment was independent of the others. The data were processed with GraphPad Prism software (version 9.5.1, CA, USA), and the experimental results are presented as the means ± standard deviations (means ± SD). Student's *t*‐test was used for comparisons between two groups, and one‐way ANOVA was used for comparisons between multiple groups. ^*^ *p* < 0.05, ^**^ *p* < 0.01, ^***^ *p* < 0.001, ^****^ *p* < 0.0001. All statistical analyses were performed using GraphPad Prism.

## Conflicts of Interest

The authors declare no conflict of interest.

## Supporting information




**Supporting File**: advs74591‐sup‐0001‐SuppMat.docx.

## Data Availability

All data pertaining to this study are accessible inside the main paper and its supplementary files. Any further inquiries for information may be addressed to, and will be answered by, the respective writers.

## References

[1] W. Sang , L. Xie , G. Wang , et al., “Oxygen‐Enriched Metal‐Phenolic X‐Ray Nanoprocessor for Cancer Radio‐Radiodynamic Therapy in Combination with Checkpoint Blockade Immunotherapy,” Advanced Science 8 (2021): 2003338, 10.1002/advs.202003338. 33643804 PMC7887592

[2] R. X. Huang and P. K. Zhou , “DNA Damage Response Signaling Pathways and Targets for Radiotherapy Sensitization in Cancer,” Signal Transduction and Targeted Therapy 5 (2020): 60, 10.1038/s41392-020-0150-x. 32355263 PMC7192953

[3] R. A. Chandra , F. K. Keane , F. E. M. Voncken , and C. R. Thomas , “Contemporary Radiotherapy: Present and Future,” The Lancet 398 (2021): 171–184, 10.1016/s0140-6736(21)00233-6. 34166607

[4] Y. Li , H. Zhang , Y. Merkher , et al., “Recent Advances in Therapeutic Strategies for Triple‐Negative Breast Cancer,” Journal of Hematology & Oncology 15 (2022): 121, 10.1186/s13045-022-01341-0. 36038913 PMC9422136

[5] M. S. Moran and A. Y. Ho , “Radiation Therapy for Low‐Risk Breast Cancer: Whole, Partial, or None?,” Journal of Clinical Oncology 40 (2022): 4166–4172, 10.1200/jco.22.01751. 36332170

[6] J. S. Vaidya , M. Bulsara , C. Saunders , et al., “Effect of Delayed Targeted Intraoperative Radiotherapy vs Whole‐Breast Radiotherapy on Local Recurrence and Survival,” JAMA Oncology 6 (2020): 200249, 10.1001/jamaoncol.2020.0249. PMC7348682 32239210

[7] Q. Yin , Y. Zhong , M. Chen , et al., “Chemotherapeutic Drug Scavenger‐Based Combination Therapy Toward Treating Triple‐Negative Breast Cancer,” Journal of Nanobiotechnology 23 (2025): 473, 10.1186/s12951-025-03571-z. 40598423 PMC12211420

[8] C. Zhou , Q. Liu , Y. Xiang , X. Gou , and W. Li , “Role of the Tumor Immune Microenvironment in Tumor Immunotherapy,” Oncology letters 23 (2022): 53, 10.3892/ol.2021.13171. 34992685 PMC8721848

[9] B. LV , Y. Wang , D. Ma , et al., “Immunotherapy: Reshape the Tumor Immune Microenvironment,” Frontiers in Immunology 13 (2022): 844142, 10.3389/fimmu.2022.844142. 35874717 PMC9299092

[10] C. Li , L. Wiseman , E. Okoh , et al., “Exploring Hypoxic Biology to Improve Radiotherapy Outcomes,” Expert Reviews in Molecular Medicine 24 (2022): 21, 10.1017/erm.2022.14. 35586915

[11] W. Bouleftour , E. Rowinski , S. Louati , et al., “A Review of the Role of Hypoxia in Radioresistance in Cancer Therapy,” Medical Science Monitor 27 (2021): e934116, 10.12659/msm.934116. 34728593 PMC8573967

[12] L. Tang , F. Wei , Y. Wu , et al., “Role of Metabolism in Cancer Cell Radioresistance and Radiosensitization Methods,” Journal of Experimental & Clinical Cancer Research 37 (2018): 87, 10.1186/s13046-018-0758-7. 29688867 PMC5914062

[13] J. Qi , F. Jin , Y. You , et al., “Synergistic Effect of Tumor Chemo‐Immunotherapy Induced by Leukocyte‐Hitchhiking Thermal‐Sensitive Micelles,” Nature Communications 12 (2021): 4755, 10.1038/s41467-021-24902-2. PMC8346467 34362890

[14] H. Deng , W. Yang , Z. Zhou , et al., “Targeted Scavenging of Extracellular ROS Relieves Suppressive Immunogenic Cell Death,” Nature Communications 11 (2020): 4951, 10.1038/s41467-020-18745-6. PMC7532538 33009382

[15] K. Qiao , Y. Huang , S. Ning , et al., “Camouflaged Nanozymes with Oxidation‐Promoting Activities Triggering Ferroptosis for Radio‐Immunotherapy,” Advanced Science 12 (2025): 2417370, 10.1002/advs.202417370. 40285563 PMC12165144

[16] Z. Deng , M. Xi , C. Zhang , et al., “Biomineralized MnO 2 Nanoplatforms Mediated Delivery of Immune Checkpoint Inhibitors with STING Pathway Activation to Potentiate Cancer Radio‐Immunotherapy,” ACS Nano 17 (2023): 4495–4506, 10.1021/acsnano.2c10352. 36848115

[17] Y. Xue , Y. Ruan , Y. Wang , P. Xiao , and J. Xu , “Signaling Pathways in Liver Cancer: Pathogenesis and Targeted Therapy,” Molecular Biomedicine 5 (2024): 20, 10.1186/s43556-024-00184-0. 38816668 PMC11139849

[18] Y. Pan , W. Tang , W. Fan , J. Zhang , and X. Chen , “Development of Nanotechnology‐Mediated Precision Radiotherapy for Anti‐Metastasis and Radioprotection,” Chemical Society Reviews 51 (2022): 9759–9830, 10.1039/d1cs01145f. 36354107

[19] H. Dong , Y. Fan , W. Zhang , N. Gu , and Y. Zhang , “Catalytic Mechanisms of Nanozymes and Their Applications in Biomedicine,” Bioconjugate Chemistry 30 (2019): 1273–1296, 10.1021/acs.bioconjchem.9b00171. 30966739

[20] X. Song , Z. Sun , L. Li , L. Zhou , and S. Yuan , “Application of Nanomedicine in Radiotherapy Sensitization,” Frontiers in Oncology 13 (2023): 1088878, 10.3389/fonc.2023.1088878. 36874097 PMC9977159

[21] M. Varzandeh , L. Sabouri , V. Mansouri , et al., “Application of Nano‐Radiosensitizers in Combination Cancer Therapy,” Bioengineering & Translational Medicine 8 (2023): 10498, 10.1002/btm2.10498. PMC10189501 37206240

[22] J. Ma , J. Qiu , and S. Wang , “Tumor Microenvironment‐responsive Nanocatalyst for Targeted Chemodynamic Cancer Therapy,” Advanced Healthcare Materials 14 (2025): 2501746, 10.1002/adhm.202501746. 40525679 PMC12391643

[23] J. Ma , J. Qiu , G. A. Wright , and S. Wang , “Oxygen/Nitric Oxide Dual‐Releasing Nanozyme for Augmenting TMZ‐Mediated Apoptosis and Necrosis,” Molecular Pharmaceutics 22 (2025): 168–180, 10.1021/acs.molpharmaceut.4c00817. 39571173 PMC11707740

[24] C. Qu , X. Shao , R. Jia , et al., “Hypoxia Reversion and STING Pathway Activation through Large Mesoporous Nanozyme for Near‐Infrared‐II Light Amplified Tumor Polymetallic‐Immunotherapy,” ACS Nano 18 (2024): 22153–22171, 10.1021/acsnano.4c05483. 39118372

[25] X. Zhu , X. Wang , Z. Liu , et al., “Peroxidase‐Like Nanozyme Activates the cGAS‐STING Pathway via ROS‐Induced mtDNA Release for Cancer Immunotherapy,” Advanced Functional Materials 34 (2024): 2401576, 10.1002/adfm.202401576.

[26] Y. Wu , Y. Zhang , J. Han , et al., “Nanozyme‐Based Biomimetic Intelligent Immune Organelles for the Treatment of Bladder‐Metastasized Tumors,” Advanced Materials 37 (2025): 11181, 10.1002/adma.202511181. 40810617

[27] J. An , Y. Yang , Y. Feng , et al., “Proton‐Driven Deformability Enables Nanozyme‐Integrated Vaccine for Enhanced Tumor Immunotherapy,” Advanced Materials 38 (2025): 09994, 10.1002/adma.202509994. 41137633

[28] Q. Ding , H. Liu , L. Yan , et al., “Engineering a Multifunctional Nanozyme Platform for Synergistic Melanoma Therapy: Integrating Enzyme Activity, Immune Activation, and Low‐Temperature Photothermal Effects,” Angewandte Chemie 137 (2025): 202505911, 10.1002/anie.202505911. PMC12322653 40454607

[29] J. Ma , J. Qiu , and S. Wang , “Nanozymes for Catalytic Cancer Immunotherapy,” ACS Applied Nano Materials 3 (2020): 4925–4943, 10.1021/acsanm.0c00396.

[30] J. Zheng , W. Meng , Z. Cui , J. Tian , and W. Zhang , “A Dual‐Enzyme‐like Photosensitive Nanozyme for Remodeling the Tumor Immunosuppressive Microenvironment to Enhance Immunotherapy,” Biomaterials 311 (2024): 122660, 10.1016/j.biomaterials.2024.122660. 38865911

[31] J. Chen , Q. Ma , M. Li , et al., “Glucose‐Oxidase like Catalytic Mechanism of Noble Metal Nanozymes,” Nature Communications 12 (2021): 3375, 10.1038/s41467-021-23737-1. PMC8184917 34099730

[32] Y. Cao , S. Ding , Y. Hu , et al., “An Immunocompetent Hafnium Oxide‐Based STING Nanoagonist for Cancer Radio‐immunotherapy,” ACS Nano 18 (2024): 4189–4204, 10.1021/acsnano.3c09293. 38193384

[33] L. Wang , H. Zhou , Q. Chen , et al., “STING Agonist‐Loaded Nanoparticles Promotes Positive Regulation of Type I Interferon‐Dependent Radioimmunotherapy in Rectal Cancer,” Advanced Science 11 (2024): 2307858, 10.1002/advs.202307858. 38063844 PMC10870073

[34] X. Wang , Y. Liu , C. Xue , et al., “A Protein‐Based cGAS‐STING Nanoagonist Enhances T cell‐Mediated Anti‐Tumor Immune Responses,” Nature Communications 13 (2022): 5685, 10.1038/s41467-022-33301-0. PMC9515186 36167857

[35] L. Hou , C. Tian , Y. Yan , L. Zhang , H. Zhang , and Z. Zhang , “Manganese‐Based Nanoactivator Optimizes Cancer Immunotherapy via Enhancing Innate Immunity,” ACS Nano 14 (2020): 3927–3940, 10.1021/acsnano.9b06111. 32298077

[36] X. Sun , Y. Zhang , J. Li , et al., “Amplifying STING Activation by Cyclic Dinucleotide–Manganese Particles for Local and Systemic Cancer Metalloimmunotherapy,” Nature Nanotechnology 16 (2021): 1260–1270, 10.1038/s41565-021-00962-9. PMC8595610 34594005

[37] C. Rébé , L. Demontoux , T. Pilot , and F. Ghiringhelli , “Platinum Derivatives Effects on Anticancer Immune Response,” Biomolecules 10 (2019): 13, 10.3390/biom10010013. 31861811 PMC7022223

[38] Q. Fu , S. Zhang , S. Shen , et al., “Radiotherapy‐Triggered Reduction of Platinum‐based Chemotherapeutic Prodrugs in Tumours,” Nature Biomedical Engineering 8 (2024): 1425–1435, 10.1038/s41551-024-01239-x. 39025943

[39] Q. Xu , Y. Zhang , Z. Yang , et al., “Tumor Microenvironment‐Activated Single‐Atom Platinum Nanozyme with H_2_ O_2_ Self‐Supplement and O_2_ ‐Evolving for Tumor‐Specific Cascade Catalysis Chemodynamic and Chemoradiotherapy,” Theranostics 12 (2022): 5155–5171, 10.7150/thno.73039. 35836808 PMC9274735

[40] S. Lu , A. Li , H. Huang , et al., “Dendrimer‐Entrapped CuPt Bimetallic Nanozymes for Tumor Microenvironment‐Regulated Photothermal/Catalytic Therapy,” ACS Applied Materials & Interfaces 17 (2025): 30716–30730, 10.1021/acsami.5c05324. 40386978

[41] J. He , T. Li , X. Pan , et al., “CD44 and αV‐Integrins Dual‐Targeting Bimetallic Nanozymes for Lung Adenocarcinoma Therapy via NIR‐Enhanced Ferroptosis/Apoptosis,” Biomaterials 323 (2025): 123407, 10.1016/j.biomaterials.2025.123407. 40403445

[42] Y. Zhang , T. Sun , and C. Jiang , “Biomacromolecules as Carriers in Drug Delivery and Tissue Engineering,” Acta Pharmaceutica Sinica B 8 (2018): 34–50, 10.1016/j.apsb.2017.11.005. 29872621 PMC5985630

[43] L. Liu , S. Fu , H. Gu , et al., “Platinum(IV)‐Backboned Polymer Prodrug‐Functionalized Manganese Oxide Nanoparticles for Enhanced Lung Cancer Chemoimmunotherapy via Amplifying Stimulator of Interferon Genes Activation,” ACS Nano 19 (2025): 2726–2741, 10.1021/acsnano.4c15115. 39772457

[44] Z. Wang , Y. Zhao , Y. Hou , et al., “A Thrombin‐Activated Peptide‐Templated Nanozyme for Remedying Ischemic Stroke via Thrombolytic and Neuroprotective Actions,” Advanced Materials 36 (2024): 2210144, 10.1002/adma.202210144. 36730098

[45] H. Zhang , J. Lv , H. Wu , et al., “Endogenous/Exogenous Dual‐Responsive Nanozyme for Photothermally Enhanced Ferroptosis‐Immune Reciprocal Synergistic Tumor Therapy,” Science Advances 11 (2025): adq3870, 10.1126/sciadv.adq3870. PMC12077522 40367177

[46] Y. Wang , H. Li , J. Lin , et al., “Engineering Nanozyme Immunomodulator with Magnetic Targeting Effect for Cascade‐Enzyodynamic and Ultrasound‐Reinforced Metallo‐Immunotherapy in Prostate Carcinoma,” Nature Communications 16 (2025): 1876, 10.1038/s41467-025-57190-1. PMC11846840 39987131

[47] J. Liu , J. Wei , S. Xiao , et al., “Multienzyme‐Activity Sulfur Quantum Dot Nanozyme‐Mediated Cascade Reactions in Whole‐Stage Symptomatic Therapy of Infected Bone Defects,” ACS Nano 19 (2025): 6858–6875, 10.1021/acsnano.4c12343. 39936642

[48] Y. Yao , R. Xu , W. Shao , et al., “A Novel Nanozyme to Enhance Radiotherapy Effects by Lactic Acid Scavenging, ROS Generation, and Hypoxia Mitigation,” Advanced Science 11 (2024): 2403107, 10.1002/advs.202403107. 38704679 PMC11234405

[49] L. Chen , M. Liu , Y. Wang , et al., “TME‐Activated MnO_2_/Pt Nanoplatform of Hydroxyl Radical and Oxygen Generation to Synergistically Promote Radiotherapy and MR Imaging of Glioblastoma,” International Journal of Nanomedicine 19 (2024): 11055–11070, 10.2147/ijn.S474098. 39502635 PMC11537150

[50] J. Wu , X. Zhu , Q. Li , et al., “Enhancing Radiation‐Resistance and Peroxidase‐like Activity of Single‐Atom COPPER Nanozyme via Local Coordination Manipulation,” Nature Communications 15 (2024): 6174, 10.1038/s41467-024-50416-8. PMC11263674 39039047

[51] Z. Liu , J. Chen , Y. Ren , et al., “Multi‐Stage Mechanisms of Tumor Metastasis and Therapeutic Strategies,” Signal Transduction and Targeted Therapy 9 (2024): 270, 10.1038/s41392-024-01955-5. 39389953 PMC11467208

[52] K. Mani , D. Deng , C. Lin , M. Wang , M. L. Hsu , and N. G. Zaorsky , “Causes of Death Among People Living with Metastatic Cancer,” Nature Communications 15 (2024): 1519, 10.1038/s41467-024-45307-x. PMC10876661 38374318

[53] W. Guo , L. Jia , L. Xie , et al., “Turning Anecdotal Irradiation‐Induced Anticancer Immune Responses into Reproducible In Situ Cancer Vaccines via Disulfiram/Copper‐Mediated Enhanced Immunogenic Cell Death of Breast Cancer Cells,” Cell Death & Disease 15 (2024): 298, 10.1038/s41419-024-06644-3. 38678042 PMC11055882

[54] C. Galassi , V. Klapp , T. Yamazaki , and L. Galluzzi , “Molecular Determinants of Immunogenic Cell Death Elicited by Radiation Therapy,” Immunological Reviews 321 (2024): 20–32, 10.1111/imr.13271. 37679959 PMC11075037

[55] A. Del Prete , V. Salvi , A. Soriani , et al., “Dendritic Cell Subsets in Cancer Immunity and Tumor Antigen Sensing,” Cellular & Molecular Immunology 20 (2023): 432–447, 10.1038/s41423-023-00990-6. 36949244 PMC10203372

[56] M. J. Pittet , M. Di Pilato , C. Garris , and T. R. Mempel , “Dendritic Cells as Shepherds of T Cell Immunity in Cancer,” Immunity 56 (2023): 2218–2230, 10.1016/j.immuni.2023.08.014. 37708889 PMC10591862

[57] S. Kim , J. H. Jeon , M. Kim , et al., “Innate Immune Responses Against mRNA Vaccine Promote Cellular Immunity through IFN‐β at the Injection site,” Nature Communications 15 (2024): 7226, 10.1038/s41467-024-51411-9. PMC11349762 39191748

[58] C. Ngo , C. Garrec , E. Tomasello , and M. Dalod , “The role of Plasmacytoid Dendritic Cells (pDCs) in Immunity during Viral Infections and Beyond,” Cellular & Molecular Immunology 21 (2024): 1008–1035, 10.1038/s41423-024-01167-5. 38777879 PMC11364676

[59] J. Zhang , M. Zha , S. Xiao , et al., “Lipid‐Coated Ag@MnO_2_ Core‐Shell Nanoparticles for Co‐Delivery of Survivin siRNA in Breast Tumor Therapy,” International Journal of Nanomedicine 20 (2025): 6515–6531, 10.2147/ijn.S510514. 40433119 PMC12106909

[60] W. Zhang , Y. Zeng , Q. Xiao , et al., “An in‐Situ Peptide‐Antibody Self‐Assembly to Block CD47 and CD24 Signaling Enhances Macrophage‐Mediated Phagocytosis and Anti‐Tumor Immune Responses,” Nature Communications 15 (2024): 5670, 10.1038/s41467-024-49825-6. PMC11227529 38971872

