# Supplementary material for: Engineering MnPt Bimetallic Nanozymes for Cascade Enzymatic Therapy and Enhanced Radio‐Immunotherapy
Source: Adv Sci (Weinh). 2026 Mar 12;13(26):e19300. doi: 10.1002/advs.202519300 (PMC13159110; doi:10.1002/advs.202519300)
Supplement: Supplementary file 1 — Supporting File: advs74591‐sup‐0001‐SuppMat.docx. [file ADVS-13-e19300-s001.docx]

Supporting Information

Engineering MnPt Bimetallic Nanozymes for Cascade Enzymatic Therapy and Enhanced Radio-Immunotherapy

Wenyi Zhang^1,2, #^, Yangyang Guo^1,2, #^, Guoping Xu^1,2, #^, Luhaoxiang Liu^1,3^, Lin Chen^1,2^, Cai Zhang^1,2^, Yiming Li^1,2^, Jianmin Li^2^, and Yang Zhao^1,2, *^

1. Department of Radiology, The Second Hospital of Tianjin Medical University, Tianjin, 300211, China.

2. Tianjin Institute of Urology, The Second Hospital of Tianjin Medical University, Tianjin, 300211, China.

3. Department of Gastroenterology, The Second Hospital of Tianjin Medical University, Tianjin, 300211, China.

*Corresponding authors: yang.zhao@tmu.edu.cn (Prof. Y. Zhao.).

# Dr. W. Zhang., Y. Guo. and G. Xu contributed equally to this work.

**Experimental section**

**Material and reagents:** Hyaluronic acid (HA), polyallylamine hydrochloride (PAH), potassium permanganate (KMnO₄), cisplatin (DDP) and Rhodamine B (RhB) were procured from Aladdin biochemical technology Co. Ltd. (Shanghai, China). 4′,6-diamidino-2-phenylindole (DAPI) was procured from Tokyo Chemical Industry Co. Ltd. (Tokyo, Japan). 3,3',5,5'-Tetramethylbenzidine (TMB) and 5,5'-Dithiobis-(2-nitrobenzoic acid) (DTNB) were purchased from Sigma‒Aldrich. All the chemicals were utilized as obtained from the manufacturer.

Western blots were performed with antibodies against of STING (19851-1-AP, proteintech, 1:2000), Phospho-STING (PA5-105674, Thermo Fisher, 1:1000), TBK1 (83686-3-RR, proteintech, 1:2000), Phospho-TBK1 (PA5-105919, Thermo Fisher, 1:1000), IRF-3 (11312-1-AP, proteintech, 1:2000), Phospho-IRF-3 (29528-1-AP, proteintech, 1:2000), β-Actin (66009-1-Ig, proteintech, 1:2000), GAPDH (60004-1-Ig, proteintech, 1:2000), HRP-conjugated Goat Anti-Mouse IgG(H+L) (SA00001-1, proteintech, 1:5000), and HRP-conjugated Goat Anti-Rabbit IgG(H+L) (SA00001-2, proteintech, 1:5000).

The antibodies used in Immunoassays include FITC-labeled anti-mouse CD11c (117305), APC-labeled anti-mouse CD80 (104713), PE-labeled anti-mouse CD86 (159203), FITC-labeled anti-mouse CD3 (100203), PE-labeled anti-mouse CD8 (100707), APC-labeled anti-mouse CD4 (100411).

**Synthesis of cationic MnO_2_**

Cationic MnO_2_ nanoparticles were synthesized via a redox reaction between KMnO_4_ and PAH under ambient conditions. Briefly, 4 mL of KMnO_4_ solution (3.5 mg/mL) was added dropwise into 10 mL of PAH solution (3.74 mg/mL) under magnetic stirring. The reaction proceeded for 15 minutes at room temperature in the dark, forming MnO_2_ nanoparticles stabilized by the cationic polyelectrolyte PAH. The resulting MnO_2_ NPs were purified by centrifugation (25,000 rpm, 30 min) and dialysis (MWCO 3.5 kDa) against deionized water for 24 hours to remove unreacted precursors.

**Synthesis of HD@MnO_2_**

10 mg of DDP and 8 mg of HA were dissolved in 10 mL of deionized water under mild stirring. The solution was then added dropwise to 5 mL of the cationic MnO_2_ NP suspension (1 mg/mL) under 500 rpm stirring for 30 minutes. The resulting HD@MnO_2_ was collected by centrifugation (12,000 rpm, 30 min), washed three times with water, and stored at 4 °C for further use.

**Characterization**

The morphology and elemental distribution were analyzed via transmission electron microscopy (TEM, Jeol, Jem-2100F). Structural analysis was carried out using X-ray diffraction (XRD) patterns, obtained with a Rigaku X-ray diffractometer (D8advance, Buker). The surface chemical state was measured via X-ray photoelectron spectroscopy (XPS, Thermo ESCALAB, 250xi). A Malvern Mastersizer 3000 was used for zeta potential and dynamic light scattering (DLS) analyses. Fourier transform infrared spectroscopy (FT-IR, VERTEX 70 spectrometer, Bruker) was applied for measurement of the infrared spectral properties. The absorption spectrum was obtained via ultraviolet‒visible spectroscopy (UV‒vis, Shimadzu UV-3600i Plus). Additionally, confocal laser scanning fluorescence microscopy (CLSM; Olympus FluoView FV1000) was used to capture high-resolution fluorescence images. The CytoFLEX S flow cytometer (Beckman) was used to collect flow cytometry data. In vivo biodistribution fluorescence images were obtained by IVIS Spectrum system (PerkinElmer). The concentration of Mn and Pt was determined by the ICP-MS (Thermo Scientific).

**Nanoparticle Stability**

HD@MnO_2_ nanoparticles were suspended in PBS buffer, 10% FBS buffer and RPMI 1640 medium at a concentration of 50 µg/mL and incubated at 37 °C with gentle shaking. Particle sizes were measured by DLS at 0, 1, 3, 5, and 7 days to assess colloidal stability.

**pH-Responsive Drug Release Assay**

To explore the Mn and DDP release behavior, the dialysis bag diffusion method was used to assess the in vitro drug release profile of the HD@MnO₂. Specifically, HD@MnO₂ (50 µg/mL) was encapsulated into a dialysis bag (molecular weight of 5000), which was sealed and immersed in the PBS buffer with different pH (5.6, and 7.4) at a volume of 10 mL. The above drug release system was placed in a constant temperature water bath at 37 °C to maintain continuous magnetic stirring. According to the set time points (1, 2, 4, 6, 8, 16, 24, 36, 48, and 72 h), 100 µL of the solution outside the dialysis bag was drawn out respectively. Finally, the withdrawn solution was detected by ICP-MS (Thermo Scientific), and the mass of Mn and Pt released by HD@MnO₂ at each time point was calculated.

**Relaxometry and MRI of HD@MnO_2_**

Proton longitudinal relaxation times were assessed by a Nuclear Magnetic Resonance System (HT-MRSI50-60KY, Shanghai, China) at room temperature. HD@MnO_2_ was suspended in PBS (H_2_O_2_^+^) or (H_2_O_2_^-^) to obtain different Mn concentrations (10‒200 µM). Longitudinal relativity (r_1_) is defined as the gradient of the linear fit for T_1_ relaxation rates (1/T_1_) in relation to Mn concentration. In vitro T_1_ FSE images were obtained with a 3.0 T MRI Scanner (MAGNETOM Vida, SIEMENS Healthineers, USA) equipped with a 32-channel phased array head coil. A standard fast spin echo sequence (FSE) was executed with the following parameters: repetition time/echo time (TR/TE) = 400/16 ms, field of view (FOV) = 160 * 160 mm^2^, slice thickness = 1.0 mm; spacing = 0.3 mm; matrix = 256 * 256, number of excitations (NEX) = 4, and bandwidth = 25 kHz.

**Enzyme-like activity measurements**

For the CAT-like activity measurements, H_2_O_2_ (100 μM) was mixed with MnO_2_ or HD@MnO_2_ (50 μg/mL, pH 5.6). Once H_2_O_2_ was added, the oxygen concentration began to be monitored by the dissolved oxygen meter. For POD-like activity measurements, H_2_O_2_ solution (100 μM) containing TMB (1 mM) was added to MnO_2_ or HD@MnO_2_ (50 μg/mL, pH 5.6) and incubated for 30 min. UV‒vis absorption spectra of the solutions at 652 nm were measured. For GSH consumption evaluation, DTNB was used as a probe, which can react with the sulfhydryl groups of GSH to form yellow 2-nitro-5-thiobenzoic acid (TNB), with a characteristic absorption peak at 412 nm. MnO_2_ or HD@MnO_2_ (50 μg/mL, pH 5.6) was mixed with GSH (10 mM) in PBS for 30 min, followed by mixing with DTNB (1 mM) for 10 min. The resulting change in the absorbance of the solution at 412 nm was recorded via a UV‒vis spectrophotometer. ·OH formation was confirmed via electron spin resonance (ESR) spectroscopy. The ·OH radical generated by MnO_2_ or HD@MnO_2_ in the presence of H_2_O_2_ was measured via an 600M spectrometer (BRUKER) with DMPO as a spin-trapping agent.

**DFT calculations**

First-principle calculations with spin polarization were conducted using the density functional theory (DFT) framework as implemented in the VASP program. The electronic exchange and correlation were treated within the generalized gradient approximation of Perdew-Burke-Enzerh. The plane wave pseudopotential method with a kinetic cutoff energy of 580 eV, based on the projector augmented wave (PAW) approach, was employed. The self-consistent total energy convergence criteria were set to be less than 10^-6^eV, and geometry optimization was terminated when the forces acting on all atoms were below 0.02 eV Å^-1^.The k-point sampling was accomplished using the Monkhorst-Pack grid method, employing 2×2×1 for geometry optimization. The free energy (G) was calculated according to Eq. 1:

G = E + Z -TS (1)

Here, E represents the total energy obtained from DFT calculations, ZPE denotes the zero-point energy, and TS corresponds to the entropic contributions.

**Molecular Docking**

Molecular docking simulations were conducted to explore the binding interaction between hyaluronic acid (HA) and the CD44 receptor. The three-dimensional structure of CD44 was retrieved from the PDB database (https://www.rcsb.org/) and prepared by repairing missing residues and assigning appropriate protonation states using AutoDockTools-1.5.6. The molecular structure of HA was obtained from the PubChem database (https://pubchem.ncbi.nlm.nih.gov/) and subjected to hydrogen addition and energy minimization using Avogadro software. Blind docking was performed with a grid box encompassing the entire protein structure. Docking calculations were carried out using the Lamarckian genetic algorithm with 20 independent runs to ensure robust sampling of binding poses and interaction energies.

**Cell Lines and Culture**

The mouse breast cancer cell line (4T1) and Human Umbilical Vein Endothelial Cell (HUVEC) were purchased from the Cell Bank of the Chinese Academy of Sciences (Shanghai, China). Mouse bone marrow dendritic cells (BMDCs) were obtained as previously described. RPMI 1640, fetal bovine serum (FBS), penicillin-streptomycin (PS), and other cell culture-related products were purchased from Gibco. 4T1 cells were cultured in RPMI 1640 supplemented with 10% FBS and 1% PS. All cells were cultured at 37°C in a humidified atmosphere containing 5% carbon dioxide (CO_2_).

**Cellular Uptake and Targeting Specificity Analysis**

MnO_2_, HA@MnO_2_ and HD@MnO_2_ for synthesizing used RhB were incubated with 4T1 cells. Cells were exposed to nanoparticles at predetermined concentrations (50 µg/mL) and time points (0, 4, 8, 12 h). Flow cytometry quantified intracellular nanoparticle fluorescence intensity across different cell types (CytoFLEX S, Beckman). Images were acquired using a confocal laser scanning microscope (CLSM, Olympus FluoView FV1000).

**Cell Viability Assay**

To evaluate tumor selectivity and biocompatibility, cell viability was assessed in 4T1 and HUVEC cell lines. Cells were seeded into 96-well plates at a density of 5000 cells/well and allowed to adhere overnight. The following day, cells were treated with MnO_2_ or [HD@MnO_2_](HA@MnO2) (50 µg/mL) at various concentrations for 24 h and then irradiated with X-ray (4 Gy, 5 Gy/min, 6 MV, VitalBeam, Varian Medical Systems). Cell viability was measured using a Cell Counting Kit-8 (Apexbio) following the manufacturer’s instructions. After adding 10 µL CCK-8 solution per well, plates were incubated for 2 h at 37 °C, and absorbance at 450 nm was recorded using a microplate reader (Varioskan Flash, Thermo Scientific). Cell viability was normalized to untreated controls. All conditions were tested in triplicate.

**ROS Assay and Calcein-AM/PI staining**

The generation of intracellular reactive oxygen species (ROS) was measured with a DCFH-DA probe (Beyotime). 4T1 cells were seeded in confocal dishes and incubated overnight. Then, the 4T1 cells were incubated with PBS, MnO_2_, or HD@MnO_2_ for 6 h. Next, the cell culture medium was replaced with fresh medium containing DCFH-DA for 20 min. For RT treatment, the cells were further exposed to X-ray irradiation (4 Gy). After X-ray irradiation, the cells were incubated for another 1 h and then stained with Hoechst (Beyotime) and imaged by confocal fluorescence imaging (CLSM). Similar to the above steps, live/dead staining was performed with a Calcein AM/PI Cell Viability Kit (Beyotime) followed by CLSM.

**Cell migration assay**

4T1 cells were seeded in a 12-well plate, and when the confluence reached approximately 85 %, a scratch was created using a sterile 10 μL pipette tip. After 0 and 24 h, observe the migration of cells under an inverted fluorescence microscope. Finally, the change in scratch area was measured to calculate the cell migration rate by imageJ software.

**Colony formation assay**

4T1 cells were seeded in 6-well plates at densities of 1000 cells per well at doses of 4 Gy. Before irradiation, MnO_2_ or HD@MnO_2_ was added to the cell culture. After treatment, the cell culture medium was changed, and the cells were cultured for 2 weeks. After culture, the cells were washed with PBS, fixed with 4% paraformaldehyde, stained with 0.2% crystal violet and photographed. The number of colonies (50–1000 cells) was counted using ImageJ software to calculate the colony formation rate.

**γ-H_2_AX, CRT and HMGB1 expression extracellular release assays**

Cells were incubated with various treatments for 24 hours, washed with PBS, and fixed with 4% paraformaldehyde. For intracellular antigens, cells were permeabilized with Triton X-100, followed by blocking of nonspecific sites with BSA. The anti-γ-H_2_AX antibody, anti-CRT antibody, or anti-HMGB1 antibody was added and incubated overnight at 4°C. After washing with PBS, the cells were incubated with fluorescently labeled secondary antibodies for 2 hours in the dark. After another wash, nuclear staining with DAPI was performed. The fluorescence signals of γ-H_2_AX, CRT and HMGB1 were observed via CLSM. Intracellular adenosine triphosphate (ATP) levels were assessed by the ATP Assay Kit. Cell supernatants and lysates were collected for detection according to the manufacturer’s protocol.

**Western Blot Analysis**

4T1 cells finished various treatment procedures, cell samples were collected and lysed in RIPA buffer containing protease and phosphatase inhibitors. Protein samples were collected from the supernatants and quantified by bicinchoninic acid (BCA) analysis. Subsequently, the equivalent protein was mixed with the protein loading buffer and boiled for 10 min. Equal amounts of each sample were applied to 10% SDS–PAGE and subjected to electrophoresis. Then, the proteins were transferred to polyvinylidene difluoride (PVDF) membranes and blocked with 5% skimmed milk buffer for 2 h at room temperature. Afterward, PVDF membranes were incubated overnight at 4 °C with primary antibodies (STING, Phospho-STING, TBK1, Phospho-TBK1, IRF-3, Phospho-IRF-3) and then secondary antibodies according to the manufacturer’s recommendations. Protein bands were visualized using enhanced chemiluminescence (ECL) and imaged using a chemiluminescence imaging system (5200, Tanon, China).

**STING Pathway Activation and Immune Cell Co-culture**

Bone marrow-derived dendritic cells (BMDCs) were isolated from femurs of BALB/c mice and cultured in RPMI-1640 supplemented with 10% FBS, 20 ng/mL GM-CSF, and 10 ng/mL IL-4 (PeproTech). 4T1 cells pretreated with HD@MnO_2_ for 24 h with RT (4 Gy) for 6 h were seeded in the lower chamber of Transwell inserts (0.4 µm pore size) at a density of 5 × 10^4^ cells/well. BMDCs and splenic T cells were co-seeded in the upper chamber at a 1:1 ratio to the tumor cells. After 48 h of co-culture, cells from both chambers were harvested for flow cytometric analysis (CytoFLEX, Beckman). BMDC maturation was assessed by staining with anti-CD80 and anti-CD86 antibodies. T cell activation was evaluated by staining with anti-CD4 and anti-CD8 antibodies. The supernatants were collected and analyzed for cytokine levels. Quantification of IFN-β, IFN-γ, IL-6, and TNF-α was performed using commercial ELISA kits following the instructions (JINGMEI BIOTECHNOLOGY).

**Tumor model establishment**

This study used 6-8 weeks female BALB/c mice purchased from the Experimental Animal Center of Tianjin Medical University. All animal experiments were approved by the Animal Ethics Committee of Tianjin University of Traditional Chinese Medicine and strictly adhered to the relevant provisions of the Regulations on the Administration of Laboratory Animals (approval number: TCM-LAEC2023104). Primary subcutaneous tumor model was established by subcutaneous injection of 4T1 cells (2×10^6^cells suspended in 100 µL saline) into mice. When tumors reached a volume of ~100 mm^3^, mice were intravenously injected with PBS, HA@MnO_2_, or HD@MnO_2_ at a dose of 1 mg/kg (n = 6). The mice needing RT treatment were subjected to 4 Gy X-ray exposure 6 h post-injection (5 Gy/min, 6 MV, VitalBeam, Varian Medical Systems). Tumor size was measured with a digital caliper every 2 days and calculated using the formula: volume = (length × width^2^) / 2. Body weights were recorded concurrently to monitor systemic toxicity. At the endpoint, mice were sacrificed, and tumors and major organs were excised for further analysis.

**Fluorescence imaging**

Luc-4T1 cells (1×10^6^) were injected into the mammary pad of mice to establish an orthotopic breast cancer primary tumor model. When the tumor volume reaches 100 mm³, the mice were randomly divided into six groups (n=6 per group). The primary tumors were treated with the drug on days 0, 2, and 4 and delivered radiotherapy (4 Gy) on days 1, 3, and 5. Tumor growth and metastasis were monitored using the IVIS system on days 7, 10, 14, 18, and 21. To minimize pain and distress, mice were anesthetized with isoflurane inhalation prior to invasive procedures. At the designated experimental endpoints, mice were euthanized by CO₂ inhalation followed by cervical dislocation to ensure death, in accordance with the approved protocol. Blood and tissue samples were collected immediately after euthanasia for further analysis.

**HE Staining**

The mice were killed after treatment, and representative heart, liver, spleen, lung, kidney, and tumor tissues were collected for histology analysis. Tissues from the mice were fixed in 10% formalin for 24 h, followed by processing and paraffin embedding. The paraffin sections were deparaffinized and stained with hematoxylin and eosin (HE). The sections were subsequently sealed with a neutral resin. The morphological changes were examined through the observation of pathological images under the brightfield microscopy (Olympus, Japan).

**Biodistribution of MnO_2_ and HD@MnO_2_ *in vivo***

Tumor-bearing mice received intravenous injections of MnO_2_ or HD@MnO_2_ at a dose of 1 mg/kg. The main organs and tumors were collected post-injection for biodistribution analysis via inductively coupled plasma atomic emission spectroscopy (ICP‒MS). Mice were sacrificed at appropriate time intervals. The organs were dissected and weighed. Next, 2 mL of HNO3 was added to each centrifuge tube fitted to the organs. And the solution was diluted to 5 mL (2% HNO3). Before the ICP-MS analysis, the standard solutions with different concentrations of 0, 10, 20, 50, 100, and 200 ppb were prepared using a 2% HNO3 solution. Both the standard and organ solutions were analyzed using ICP-MS. The amount of Mn was normalized to the tissue weight per gram.

**Cytokine Quantification by ELISA**

Tumor tissues were homogenized in cold PBS containing protease inhibitors and centrifuged at 12,000 × g for 15 minutes at 4 °C. The supernatants were collected and analyzed for cytokine levels. Quantification of IFN-β, IFN-γ, and TNF-α was performed using commercial ELISA kits (JINGMEI BIOTECHNOLOGY) following the manufacturer’s instructions. Briefly, samples and standards were added in duplicates to antibody-coated 96-well plates, incubated with detection antibodies, followed by streptavidin-HRP and TMB substrate. After stopping the reaction, absorbance was measured at 450 nm, and cytokine concentrations were calculated using standard curves. All procedures were performed at room temperature unless otherwise stated.

***In vivo* MRI**

The efficiency of T1-weighted MR signal enhancement on tumor-bearing mice was monitored using MRI (MAGNETOM Vida, SIEMENS Healthineers, USA). Before MR, tumor-bearing mice were anesthetized with isoflurane and bundled. Subsequently, HD@MnO_2_ was administered via the tail vein. Dynamic T1 imaging was conducted before and at certain times following injection with a 3.0 T MRI Scanner System carried with a special coil for small animal imaging. The imaging settings employed with an FSE sequence were: repetition time/echo time (TR/TE) = 400/15 ms, field of view (FOV) = 16 × 16 cm^2^, slice thickness (SLT) = 1.0 mm; spacing = 0.5 mm; matrix = 512 × 256; NEX = 4 and bandwidth = 25 kHz. To further observe the enhanced effect of the NPs, the signal intensity of the regions of interest (ROIs) at each imaging time point was measured three times by hand. The MRI signal intensity was measured using RadiAnt DICOM Viewer 2023.1.

**Establishment of a lung colonization model**

Each mouse was injected with Luc-4T1 tumor cells (100 µL, 2 × 10^5^) through the tail vein. After 5th days, the mice were randomly divided into six groups (n = 3 per group), and the lungs were treated with the X-ray on the 1st, 3rd and 5th days after tail vein injection (1 mg kg^−1^). The tumor metastasis of mice was observed by the IVIS system on the 14th and 28th days, respectively. The lung metastasis tumor-bearing mice were sacrificed on day 35th, lungs were harvested and kept in Bouin’s fixative solution overnight to detect metastasis. The pulmonary metastatic nodules were counted and analyzed. Lung tissues were embedded and sectioned for further hematoxylin and eosin assay.

**Statistical Analysis**

All the experimental data were repeated three or more times, and each experiment was independent of the others. The data were processed with GraphPad Prism software (version 9.5.1, CA, USA), and the experimental results are presented as the means ± standard deviations (means ± SD). Student’s t-test was used for comparisons between two groups, and one-way ANOVA was used for comparisons between multiple groups. *p < 0.05, **p < 0.01, ***p < 0.001, ****p < 0.0001. All statistical analyses were performed using GraphPad Prism.

**Supplementary Tables**

**Table S1. Mn and Pt loading rate of HD@MnO_2_**

|  | Mn (yield) | Pt (yield) |
| --- | --- | --- |
| MnO_2_ | 71.79±1.66% | — |
| MnO_2_-DHzyme | 72.80±2.10% | 63.62±1.06% |

**Supplementary Figures**

**
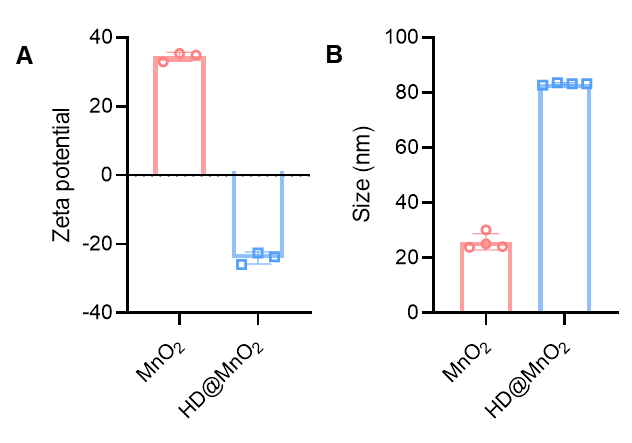
**

**Figure S1.** A) Zeta potential of MnO_2_ and HD@MnO_2_. B) Diameter distribution of HD@MnO_2_.


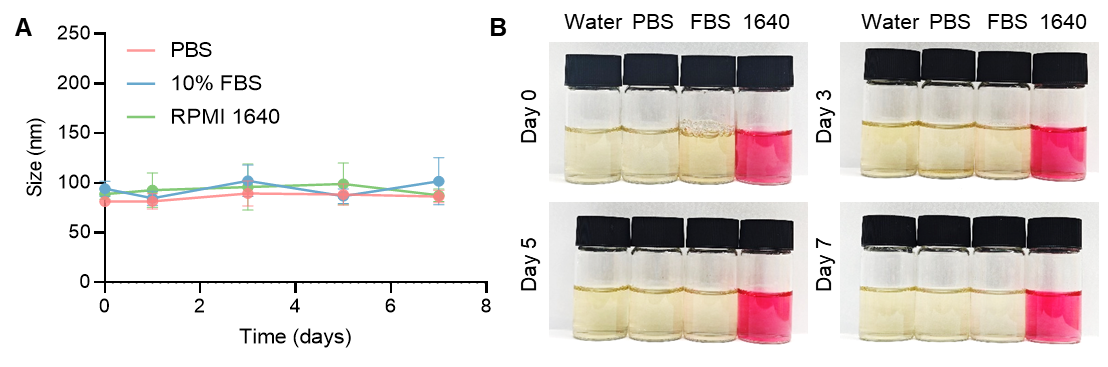


**Figure S2.** A) The variation of HD@MnO_2_ particle size over a week during incubation in PBS buffer, 10% FBS buffer and RPMI 1640 medium. B) The photos of HD@MnO_2_ dispersed in various media for 0, 3, 5 and 7 days.


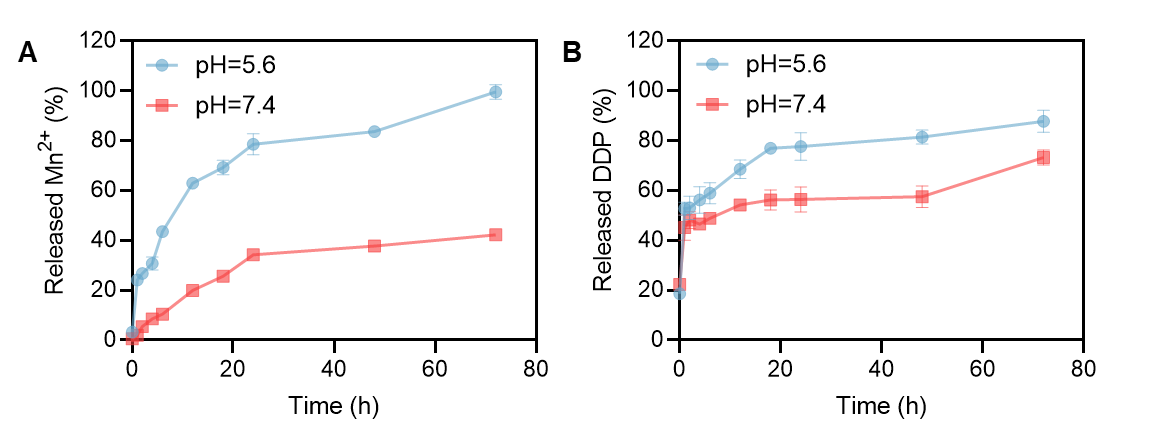


**Figure S3.** A) In vitro release profiles of Mn^2+^ under pH 7.4 and 5.6. B) In vitro release profiles of DDP under pH 7.4 and 5.6.


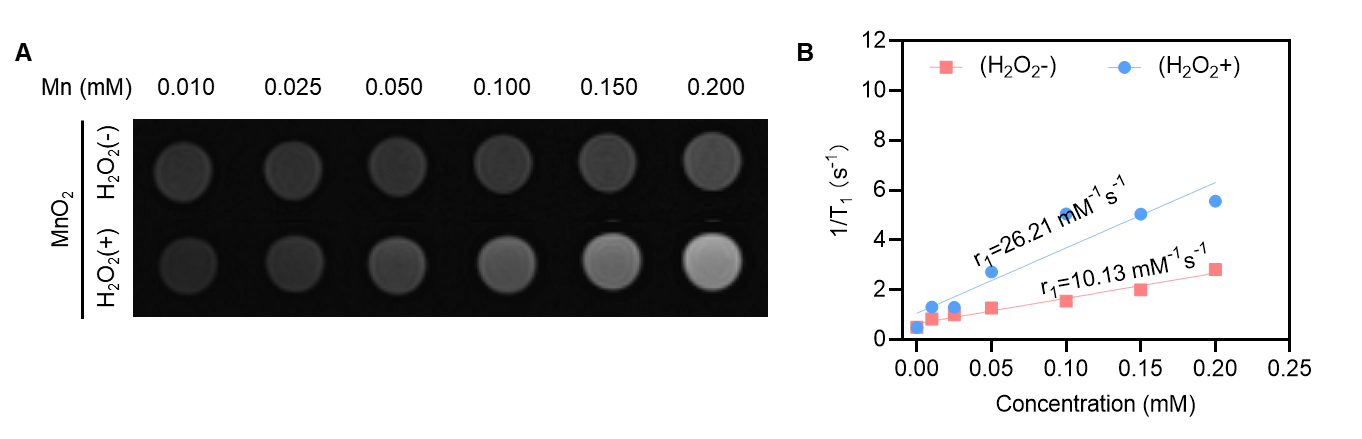


**Figure S4.** A) T1-weighted MRI images of cationic MnO_2_. B) The longitudinal (r_1_) relaxation rates acquired from cationic MnO_2_.

**Figure S5.** Dissolved oxygen curves under different treatment conditions.


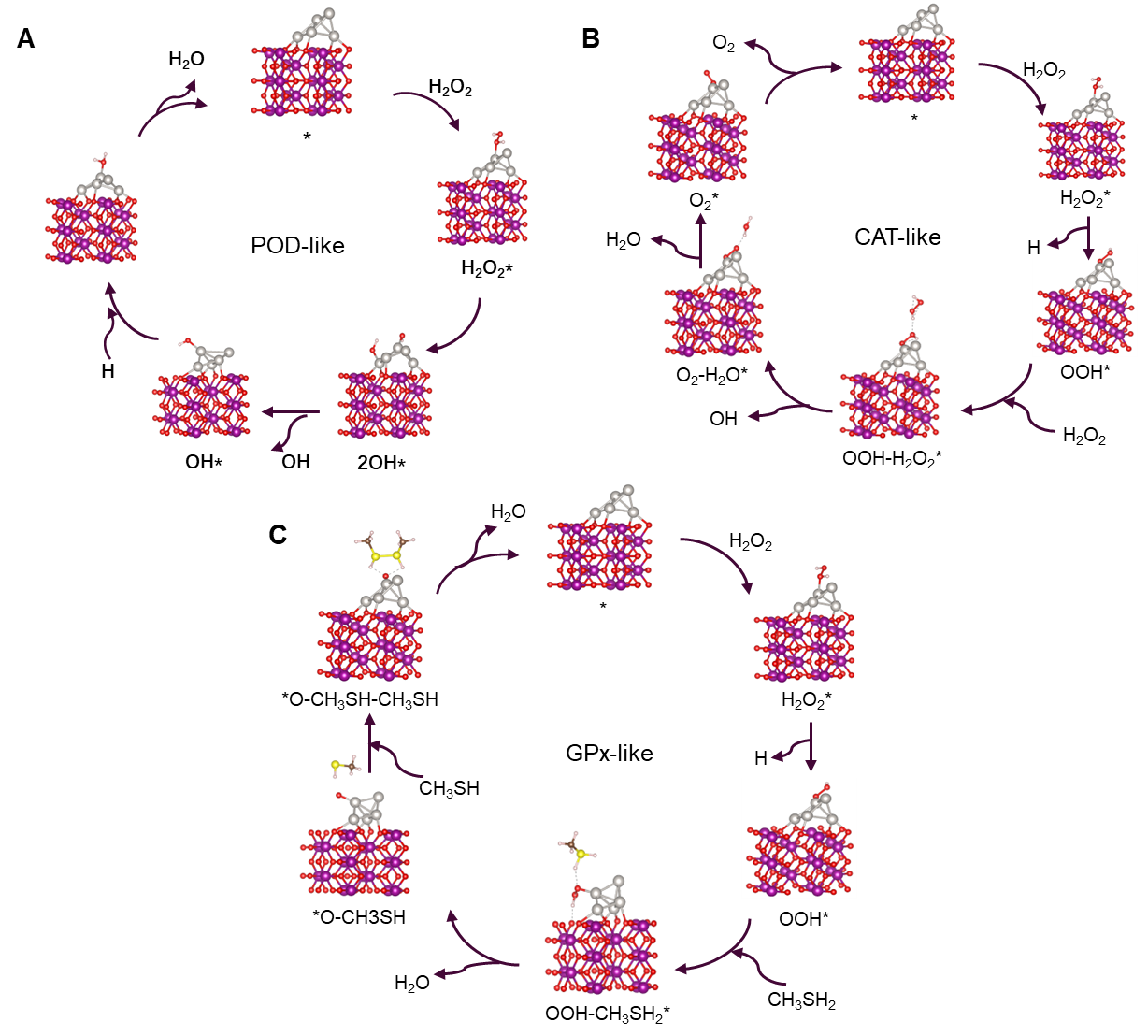


**Figure S6.** A) POD-like pathways of HD@MnO_2_. B) CAT-like pathways of HD@MnO_2_. C) GPx-like pathways of HD@MnO_2_.


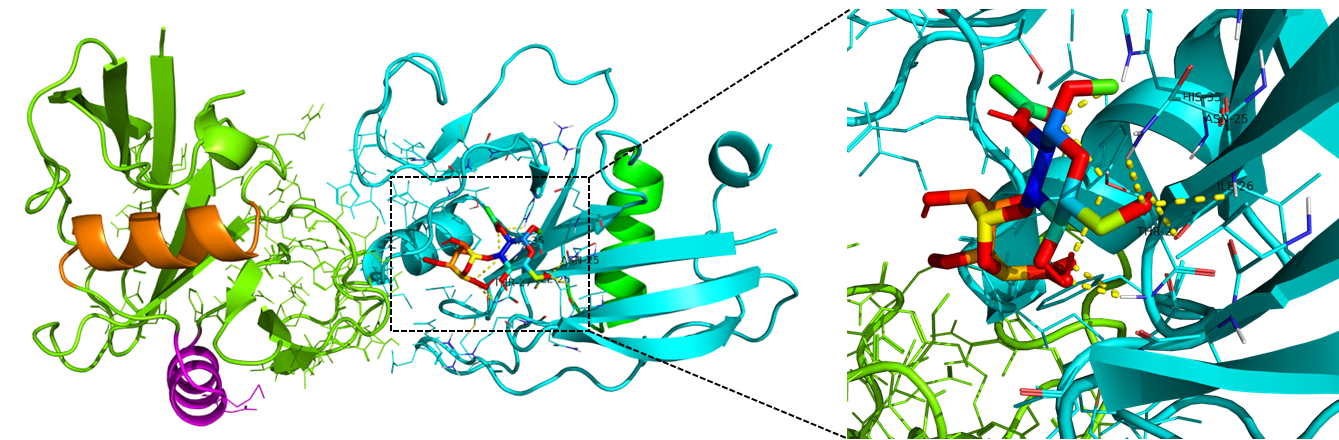


**Figure S7.** Molecular docking between HA and CD44 receptor.


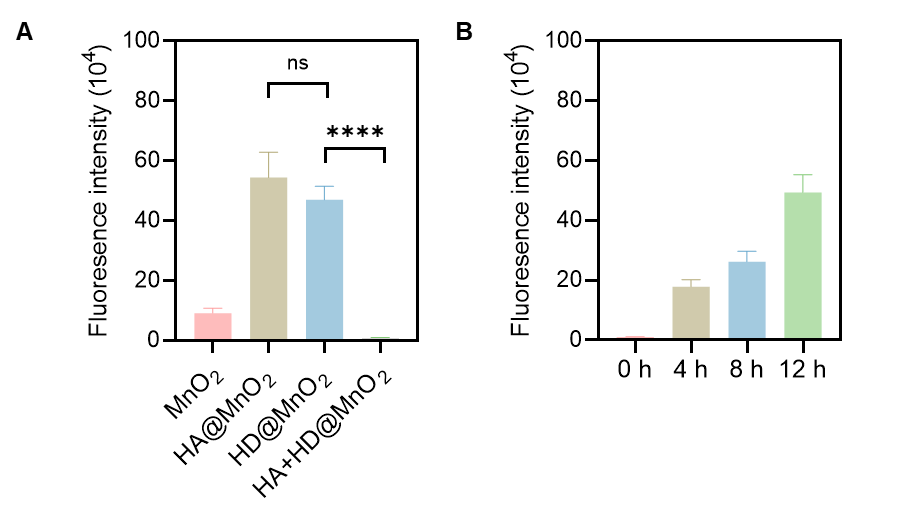


**Figure S8.** A) Quantification of fluorescence intensity of Figure 3A. B) Quantification of fluorescence intensity of Figure 3B. The data are presented as the means ± SD, n = 3, *P < 0.05, **P < 0.01, ***P < 0.001, and ****P < 0.0001. NS, no significance difference.

**Figure S9.** ATP release by 4T1 cells after different treatments. The data are presented as the means ± SD, n = 3, *P < 0.05, **P < 0.01, ***P < 0.001, and ****P < 0.0001. NS, no significance difference.

**
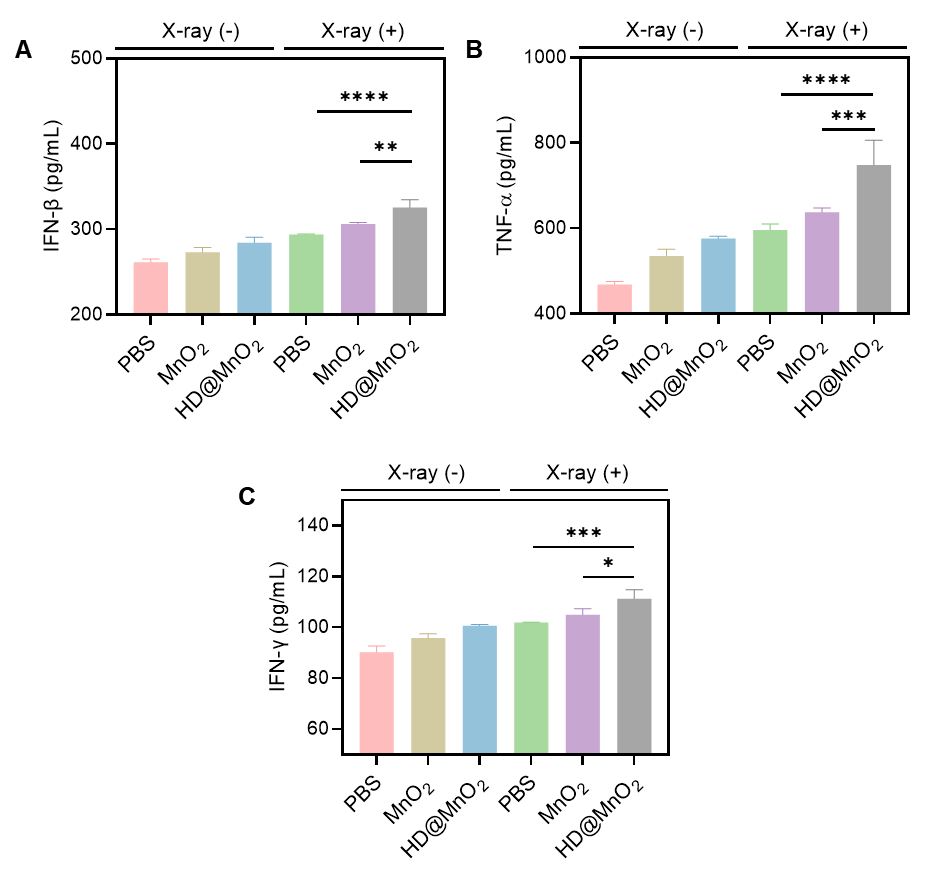
**

**Figure S10.** Cytokine concentrations of IFN-β A), TNF-α B), and IFN-γ C) in supernatants after indicated treatments. Data presented as mean ± S.D. (n = 3). *P < 0.05, **P < 0.01, ***P < 0.001, ****P < 0.0001.


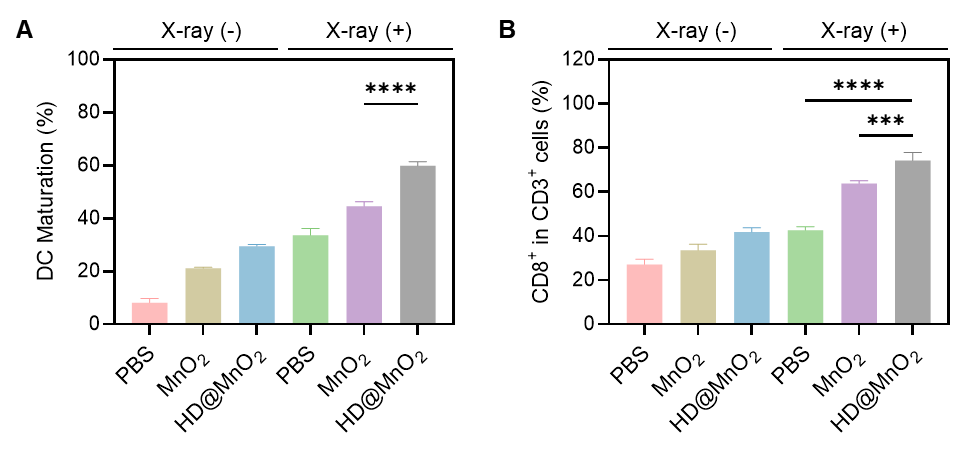


**Figure S11.** A) The corresponding quantitative analysis of BMDCs maturation induced by culture medium supernatant of 4T1 cells. B) CD8^+^ T cells were induced by culture medium supernatant of 4T1 cells and the corresponding quantitative analysis pretreated with different treatments. Data presented as mean ± S.D. (n = 3). *P < 0.05, **P < 0.01, ***P < 0.001, ****P < 0.0001.


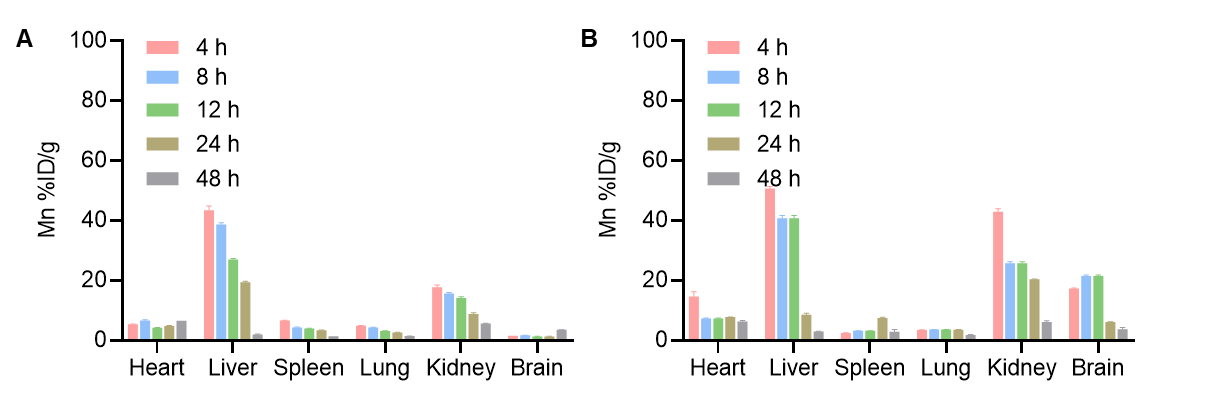


**Figure S12.** Biodistribution of intravenous HD@MnO₂ A) and MnO₂ B) at 4 hours, 8 hours, 12 hours, 24 hours and 48 hours post-administration (n = 3).


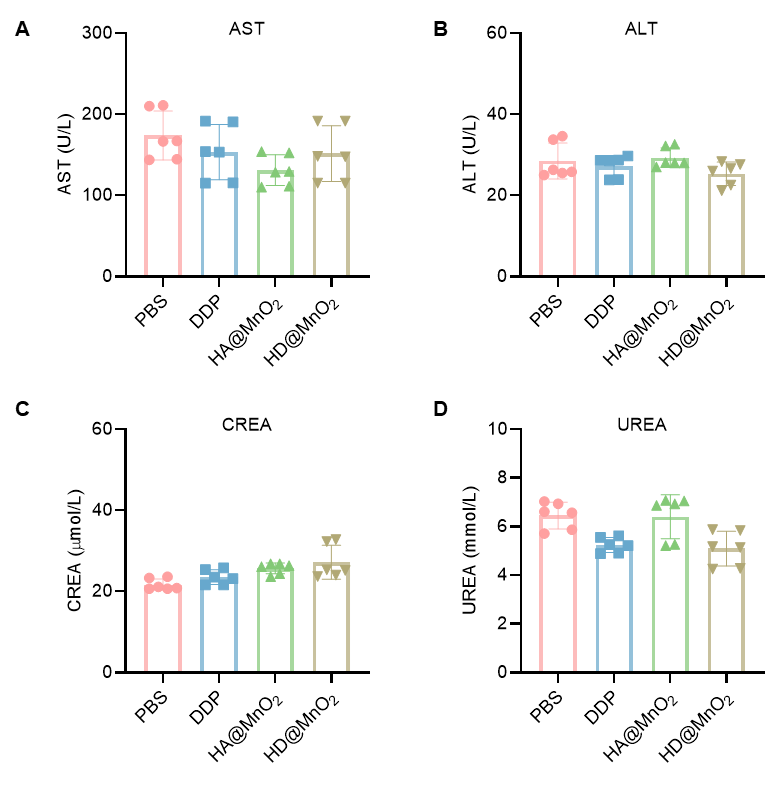


**Figure S13.** Biosafety evaluation. (A-D) Routine blood and biochemical analyses of the liver and kidney (n=6).


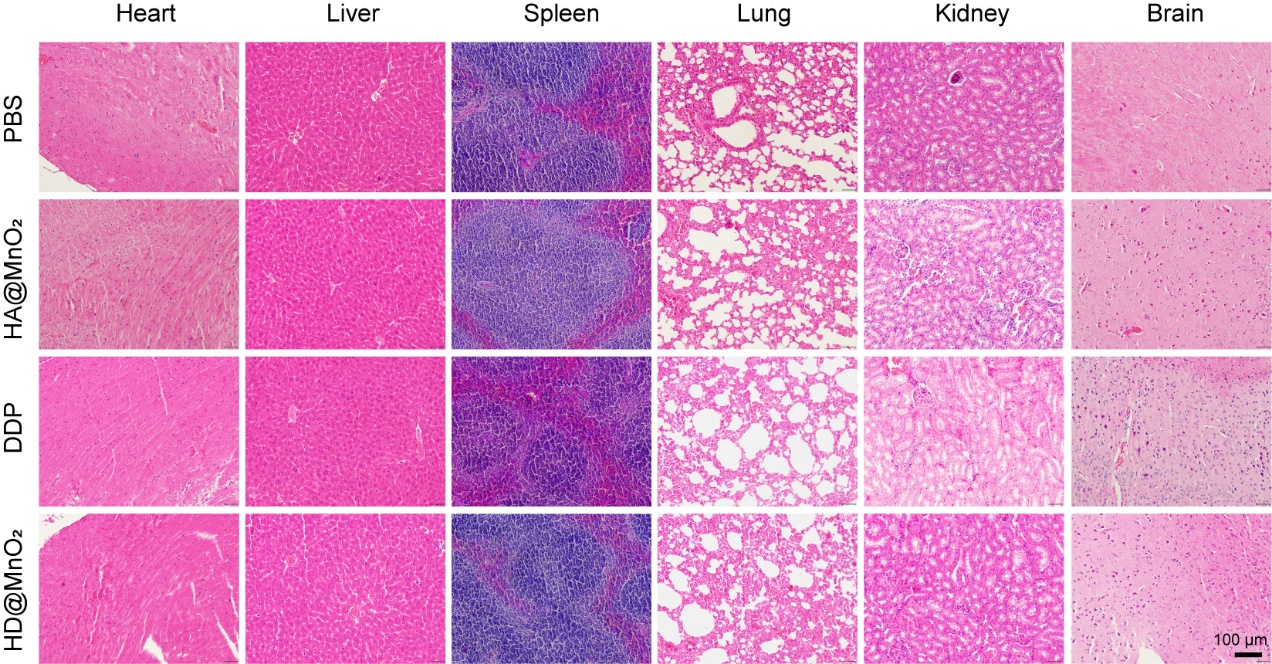


**Figure S14.** HE staining of main organs from mice that received various treatments (scale bar: 100 µm).


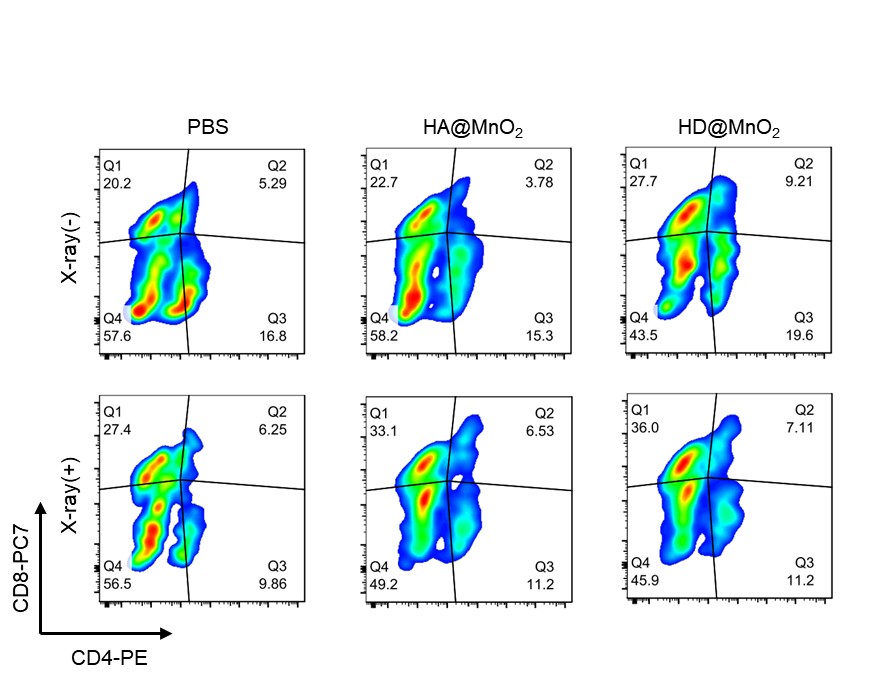


**Figure S15.** Flow cytometry analysis of CD8^+^ T cells and CD4^+^ T cells in tumors after various treatments (n = 3).

**
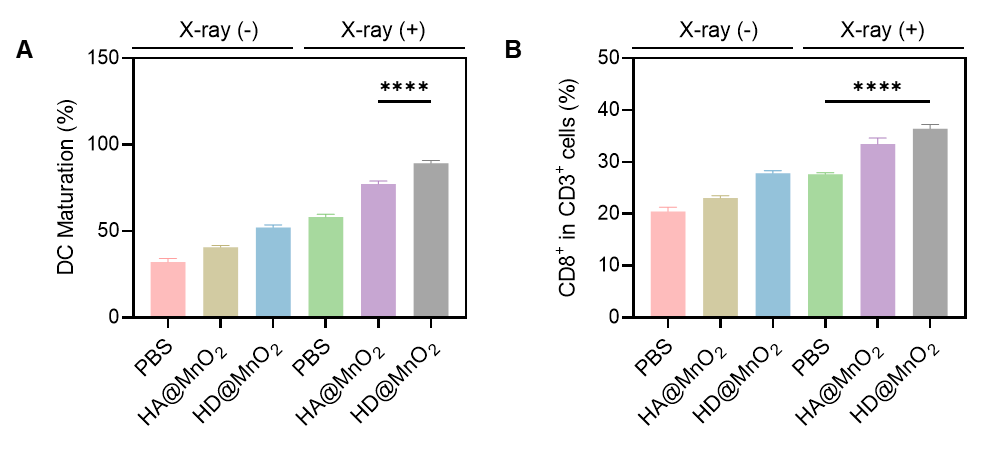
**

**Figure S16.** A) Quantitative analysis of matured (CD80^+^ CD86^+^) DCs in tumors after treatments. B) Quantitative analysis of CD8^+^ T cells and CD4^+^ T cells in tumors after treatments. The data are presented as the means ± SD, n = 3, *P < 0.05, **P < 0.01, ***P < 0.001, and ****P < 0.0001. NS, no significance difference.
